# Cardiac macrophage subsets differentially regulate lymphatic network remodeling during pressure overload

**DOI:** 10.1038/s41598-021-95723-y

**Published:** 2021-08-19

**Authors:** Mathilde Bizou, Romain Itier, Mina Majdoubi, Dounia Abbadi, Estelle Pichery, Marianne Dutaur, Dimitri Marsal, Denis Calise, Barbara Garmy-Susini, Victorine Douin-Echinard, Jérome Roncalli, Angelo Parini, Nathalie Pizzinat

**Affiliations:** 1 I2MC, Toulouse University, Inserm, Université Paul Sabatier, Toulouse, France; 2 Department of Cardiology, INSERM U1048-I2MC, CARDIOMET, University Hospital of Toulouse, Toulouse, France; 3 UMS006-Microsurgery Facility, Toulouse, France; 4 INSERM UMR-1048, Institut de Médecine Moléculaire de Rangueil, Bât L3, CHU Rangueil 1, Av. J. Poulhès, 31403 Toulouse Cedex 4, France

**Keywords:** Cardiology, Cardiovascular biology

## Abstract

The lymphatic network of mammalian heart is an important regulator of interstitial fluid compartment and immune cell trafficking. We observed a remodeling of the cardiac lymphatic vessels and a reduced lymphatic efficiency during heart hypertrophy and failure induced by transverse aortic constriction. The lymphatic endothelial cell number of the failing hearts was positively correlated with cardiac function and with a subset of cardiac macrophages. This macrophage population distinguished by LYVE-1 (Lymphatic vessel endothelial hyaluronic acid receptor-1) and by resident macrophage gene expression signature, appeared not replenished by CCR2 mediated monocyte infiltration during pressure overload. Isolation of macrophage subpopulations showed that the LYVE-1 positive subset sustained in vitro and in vivo lymphangiogenesis through the expression of pro-lymphangiogenic factors. In contrast, the LYVE-1 negative macrophage subset strongly expressed MMP12 and decreased the endothelial LYVE-1 receptors in lymphatic endothelial cells, a feature of cardiac lymphatic remodeling in failing hearts. The treatment of mice with a CCR2 antagonist during pressure overload modified the proportion of macrophage subsets within the pathological heart and preserved lymphatic network from remodeling. This study reports unknown and differential functions of macrophage subpopulations in the regulation of cardiac lymphatic during pathological hypertrophy and may constitute a key mechanism underlying the progression of heart failure.

## Introduction

Heart failure (HF) is a leading cause of mortality worldwide and represents the end stage of cardiac diseases from different etiologies such as myocardial infarction or more long-standing diseases such as pressure and volume overload. Recently the lymphatic system has emerged as an important regulator of the interstitial fluid compartment and cardiac remodelling during pathology. The experimental obstruction of cardiac lymphatics led to cardiac edema and ventricle dysfunction. This situation was associated to myocardial stiffness and fibrosis by increasing the amount of collagen content in the ventricle walls^1–3^. More recently dysfunctional lymphatic promoted exacerbation of chronic inflammation and long-term deterioration of cardiac function after myocardial infarction^4^. Inversely, stimulation of lymphangiogenesis by VEGFc treatment after myocardial infarction was found to reduce fibrosis and inflammation and to improve cardiac function^5,6^. On the other hand, in a model of minor antigen (HY) sex-mismatched murine heterotopic cardiac transplantation, the accumulation of transplanted donor graft cells in the draining lymph nodes of recipient mouse were associated with an enhanced lymphatic flow and a myocardial lymphatics increase. Thus lymphangiogenesis may facilitate migration of antigen-presenting cells and chronic graft rejection^7^.

Several studies have suggested that macrophages play active roles in lymphatic growth during tumour expansion and embryonic development^8–10^*.* In parallel others studies have highlighted the involvement of macrophages in the heart failure development or maintenance^11–13^. Indeed, monocytes/macrophages have been described as a major group of leukocytes infiltrating the injured hearts where they contribute in exacerbating pathological cardiac remodeling in concert with others immune cells, such as CD4^+^ T-cells^14–17^. They also take part in the process of healing and restoring tissue homeostasis with potential contributions from regulatory T-cells^18,19^.

Macrophages coexist as distinct populations, resident macrophages that originate from erythro-myeloid precursors persists into adulthood by means of local self-renewal in different tissues including the heart and the peritoneal cavity^20^. In addition, during cardiac pathology and over time, CCR2^+^ monocyte-derived macrophages predominantly replaced resident macrophages^18^. These monocyte-derived macrophages recruited into the murine heart during acute injury, lack regenerative proprieties observed for resident macrophages^21,22^. The lack of selective cell-surface markers complicates the comprehension of macrophage subsets contribution in inflammatory processes, tissue homeostasis and adaptation to stress^23^. To date the involvement of different macrophage subpopulations in cardiac lymphatic remodeling is still poorly defined. LYVE-1 (Lymphatic vessel endothelial hyaluronic acid receptor 1), a transmembrane receptor, was initially identified as a selective marker for lymphatic vessels. LYVE-1 serves as a docking receptor for hyaluronic acid -coated leukocytes that triggers cell entry to peripheral lymphatics and their migration to downstream lymph nodes through recognition of chemokines gradients by immune cell cognate receptors^24,25^. Besides, the expression of LYVE-1 was observed in macrophage subpopulations present in a broad range of tissues including heart, lung, and arteries^26–29^. This subset was also associated with the lymphatic vessels during embryogenesis and tumour development^30^.

In the present study, we observed a remodeling of lymphatic network and a decrease of lymphatic efficiency occurring after TAC (transverse aortic constriction) induced heart failure. The number of cardiac endothelial cells was correlated with a subset of cardiac macrophage identified by LYVE-1 expression and with the ventricular systolic function of mice. This macrophage subset that expressed pro-lymphangiogenic factors sustained lymphangiogenesis in vitro and in vivo*.* In contrast, their counterpart, negative for LYVE-1 that appeared replenished by CCR2^+^ monocytes during TAC, altered lymphatic endothelial LYVE-1 receptors. Modification of macrophage population ratio obtained by blocking CCR2^+^ monocytes recruitment during pressure overload attenuated the cardiac lymphatic remodeling. This study reports an unknown and differential regulation of the lymphatic network by cardiac macrophage subpopulations during chronic pressure overload.

## Methods

### Animals, transverse aortic constriction and echocardiography

Mice in a C57BL/6 background were obtained from Janvier–France. 12-weeks-old male mice were used in all the experiments and were maintained under specific pathogen–free conditions. Mouse experiments were approved by the Animal Care and Use Committee of the University of Toulouse, INSERM/ENVT (agreement number: C3155507) and were conformed to the guidelines for ethical care of experimental animals of the European Union (2010/63/EU). All experimental procedures are in accordance with ARRIVE guidelines. Male mice were anesthetized with intraperitoneal ketamine (60 mg/kg) and xylazine (6 mg/kg) injection. Anesthesia was maintained during surgical procedure with isoflurane (2%). Mice were ventilated, and the left thorax was opened in the second intercostal space. Aortic constriction was performed by ligating the transverse aorta using a 7-0 Prolene suture under a dissecting microscope as previously described^15^. A subcutaneous injection of buprenorphine (Buprecar—100 µg/kg) was administrated to mice for pain relief. Age-matched animals underwent identical surgical procedure, except for ligation of the aorta (SHAM-operated mice). For CCR2 antagonism, mice were daily sub-cutaneously injected with 2 mg/kg RS504393 (Tocris Bioscience) or vehicle. After 1 or 6 weeks of TAC, echocardiography was performed on isoflurane anesthetized mice and ventricular dimensions were measured from 2-dimensionally guided M-mode echocardiograms. The echocardiographer was blinded to mouse treatment. Heart collection was performed on pentobarbital anesthetized animals after in situ PBS perfusion to wash out blood from ventricular cavities and coronary arteries.

### Cardiomyocyte isolation and phagocytosis assays

Adult mouse cardiomyocytes from 10 to 12 week-old mice were isolated using a Langendorff apparatus as previously described^17^. Briefly, excised heart from anesthetized mice was rapidly mounted on a Langendorff apparatus and perfused for 20 min with low-calcium solution (LCS) prewired at 37 °C [117 mmol/L NaCl, 5.7 mmol/L KCl, 4.4 mmol/L NaHCO_3_, 1.5 mmol/L KH_2_PO_4_, 1.7 mmol/L MgCl_2_, 11.7 mmol/L d(+)-glucose, 21 mmol/L HEPES, 20 mmol/L taurine, 10 mmol/L creatine (pH 7.2)]. The solution was then quickly changed to LCS plus 1 mg/mL collagenase type I (Worthington Biochemicals), and 1 mg/mL albumin bovine for 10 min. The heart was minced and stirred in LCS. Cardiomyocytes present in the supernatant were purified by gravity sedimentation, collected, and culture overnight in a laminin coated dish in the absence of serum. Twenty four hours later, most of the cells were apoptotic or necrotic. They were labeled with CFSE (2.5 µg/mL CFSE, for 15 min at 37 °C) and incubated with freshly isolated macrophages. Alternatively purified cardiomyocytes were immediately frozen and RNA extracted as described below.

Cardiac CD45 positive cells were isolated from adult mouse heart using positive kit selection CD45 MACS separation (MiltenyiBiotec) and plated on culture dish for 2 h, non-adherent cells removed and then 1 mg/mL of bioparticles (pHrodo™ Red E. coli BioParticles™ Conjugate, Life Technologies) was added for 1 h in 37 °C or 4 °C in order to distinguish active phagocytosis from cell surface binding. In a different set of experiment cultured cardiomyocytes labeled with CFSE (2.5 µg/mL CFSE, for 15 min at 37 °C) were added in ratio 10 to 1 with freshly isolated and adherent CD45^+^ cells for 2 h. Then, all cells were collected and stained with anti-CD11b, anti-CD64, anti-LYVE-1 and anti-MHCII to identify macrophage subsets positive for CFSE or pHrodo™ BioParticles. Live/dead Yellow fluorescent reactive dye (Invitrogen) was used to determine cell viability. Data were analyzed by DIVA software. CFSE labeled phagosomes of isolated and adherent CD45^+^ cells formed after 2 h incubation were also visualized after immunostaining with anti-Lyve-1 and images were acquired by a Zeiss Microscope.

### Lymphangioma and cardiac macrophages, cell isolation and culture

A mouse lymphangioma model was produced according to Mancardi et al.^31^. Benign lymphangiomas were induced by two intra-peritoneal injections of Freund’s incomplete adjuvant (FIA, sigma) within a 2-week interval. FIA was mixed with an equal volume of phosphate- buffered saline (PBS) and 0.2 mL was injected into 10 to 12-week-old C57Bl6 mice. Lymphangiomas were present 2 weeks after the last injection and were mostly associated with the diaphragm. Tissue was removed, digested with 50 µg/mL of Liberase™ (Sigma) and submitted to FACS sorting. Cells were stained with directly conjugated Abs (see Table 1 in supplementary file). For gating strategy, cells were first identified on a forward scatter/side scatter (FSC-A/SSC-A) dot plot, the doublets were removed and live cells were selected. Then, these cells were characterized by the expression of CD45 (leukocyte). After removing Ly6G^+^ cells, macrophages were identified as CD11b^+^ and CD64^+^ cells, the different macrophage subsets were discriminated by LYVE-1 and MHCII expression. Within CD45 negative population, the expression of CD31 and LYVE-1 was used to distinguish between blood endothelial cells (CD45^−^CD31^+^LYVE-1^−^) and lymphatic endothelial cells (CD45^−^CD31^+^LYVE-1^+^). Countbright beads were selected by their FSC-A/SSC-A parameters and were used to normalize cell acquisition numbers. Viable cells, sorted on Fluorescence-Activated Cell Sorting procedure, were immediately frozen or cultured in SFM medium (Thermo Fisher). Total RNA was extracted from frozen cells using ReliaPrep™ (Promega) and reverse transcription was performed on 0.1 µg RNA using SuperScript VILO (Invitrogen, Life Technologies). For cell culture, FACS sorted cells L^+^ and L^−^MHCII^hi^ (L^−^) (200,000 for lymphangioma and 100,000 for cardiac tissue) were seeded in 96 well plates for 2–3 h with Serum-Free Media (SFM) (Thermo Fisher), then unattached cells were removed and cultured in SFM for 48 h to constitute macrophage conditioned medium.

### Real time PCR analysis

RNA was extracted using the Qiagen RNeasy Mini Kit from cardiac tissue, according to the manufacturer’s instructions. DNase treatment was systematically performed. Quality and quantity of extracted RNA were assessed by Experion analysis. The cDNA was synthesized using Superscript II First-Strand system or RT-Vilo (Invitrogen). The absence of contaminants was checked by RT-PCR assays of negative control samples in which the Superscript II was omitted. mRNA was analyzed by real-time PCR using SYBR green (Takara) probe method and the primers listed in Table 1. Melting curve analysis was performed to ensure purity of the PCR products and relative quantification was calculated using the comparative cycle threshold (CT) method (2−∆∆CT) with data normalized to housekeeping genes 36B4 and HPRT and calibrated to the average of control group (SHAM unless otherwise notified).

### Lymphatic endothelial cells culture

Human dermal lymphatic endothelial cells (HDLEC) were provided by Promocell and were cultured in Endothelial Growth Medium (EGM2, Promocell supplemented with growth factors). The cells retain lymphatic endothelial characteristics and functionality over at least 7 passages. Tube formation was assayed on growth factor reduced Matrigel (Corning Inc). 120 µl of Matrigel were added in 48-well plates and allowed to polymerize for 2 h at 37 °C. After trypsinization, HDLECs were seeded on the gel at a density of 3 × 10^4^ cells/well and incubated with the conditioned medium of L^−^ or L^+^ macrophages or control media. Preliminary experiments were performed to examine the responses of the LEC to different dilutions (1/2, 1/4, 1/6, 1/10) of macrophages conditioned medium obtained from 200 000 FACS lymphangioma sorted L^+^ or L^−^ cells and cultured in Serum-Free Media (SFM) (Thermo Fisher) for 48 h. Maximal responses on the formation of three-dimensional capillary-like structures were obtained with a 1/6 dilution of macrophage conditioned media into EGM2 without growth factor supplementation, therefore this dilution was consequentially used in all experiments using macrophage conditioned media unless otherwise indicated. Capillary-like structures organized by the cells were viewed and photographed 4 h after incubation. The branching point and the number of closed tubes or loops were quantified by ImageJ software (National Institutes of Health). Scratch assays were performed in a 24-well plate seeded with 5 × 10^4^ HDLEC/well. As soon as the cells reached confluence, a lesion was generated in a standardized fashion using a 200 µL pipette tip. The medium was replaced by 500 µL of diluted macrophage L^−^ or L^+^ conditioned media or SFM as a negative control and cultivated for 15 h at 37 °C under 5% CO_2_. Images of the lesion were immediately captured (T0) and after 15 h. ImageJ was used to measure the wound size at T0 and after 15 h. Migration distance was obtained by subtracting wound size after 15 h from and expressed in µm. To identify the factors with biological effect on HDLEC, inhibitor of VEGFR3 (SAR131675 100 nM), FGFR1 (PD173074 100 nM) or IGF-1R (PPP 0.5 µM) purchased from Bertin Bioreagent were added in macrophage conditioned media and incubated with HDLECs. In addition HLECs treated with diluted L− or L+ macrophage conditioned media were collected after 36 h and LYVE-1 expression was evaluated by Western blot and RT-PCR.

### Transfection of CHO cells, cleavage of LYVE-1 protein and immunodetection

CHO cells were transfected with murine LYVE-1 cDNA (Sinobiological) using the Transfection Reagent X-tremeGENE™ (Sigma-Aldrich), then cells were selected by resistance to Hygromycin (50 μg/mL Invivogen). Conditioned media of lymphangioma and heart isolated L− and L+ or 400 ng of recombinant MMP12 (Biolegend) were preincubated with 30 µM p-aminophenylmercuric acetate at 37 °C for 30 min, then incubated with LYVE-1 transfected CHO cells for 12 h. At the end of incubation period, the cells were rinsed with cold PBS and detection of LYVE-1 present on live CHO cell surface was realized by flow cytometry with the anti-LYVE-1 antibody (ALY7- ebiosciences).

Confluent monolayers of HLEC were incubated with lymphangioma isolated L^−^ macrophage conditioned media into EGM2 for 36 h in presence or absence of 10 µM Batimastat. Recombinant human MMP12 obtained from Biolegend was first activated by preincubation with 40 µM p-aminophenylmercuric acetate in PBS (Sigma-Aldrich) for 1 h at 37 °C. Then 200 ng of activated MMP-12 or control medium containing only 40 µM p-aminophenylmercuric acetate was incubated for 5 h on a confluent monolayer of HLEC at the concentration of 1 ng/µL. At the end of incubation period, the cells were rinsed with cold PBS and proteins were extracted.

Protein quantification by capillary Western System (JESS, ProteinSimple). HLEC were lysed using lysis buffer (Tris HCL 50 mM, NaCl 150 mM, NP40 1%, Deoxycholic Acid 1%, SDS 0.10%, EDTA 5 mM) supplemented with protease and phosphatase inhibitor cocktails (Sigma). Protein concentrations were determined using a BCA Protein Assay Kit (Thermo Fisher Scientific) and adjusted to 0.2 mg/mL. Proteins were separated using JESS Separation Capillary Cartridge 12–230 kDa and LYVE-1 protein detected with the rabbit anti human LYVE-1 antibody (HPA042953 Atlas antibodies 1/50) according to the manufacturer and normalized to total protein content by protein normalization buffer. The signals generated were detected and analyzed by Compass software (ProteinSimple). For MMP12 immunodetection, equivalent quantity of proteins from 200,000 L^+^ and L^−^M^hi^ (L^−^) lymphangioma cells sorted by FACS were separated using JESS Separation Capillary Cartridge 12–230 kDa and revealed with anti MMP12 antibody (R&D).

### Cardiac Injection of macrophage subsets

C57Bl/6 WT mice were anesthetized with intraperitoneal ketamine (60 mg/kg) and xylazine (6 mg/kg) injection. Anesthesia was maintained during surgical procedure with isoflurane (2%), 200,000 lymphangioma L^−^ or L^+^ macrophages isolated by FACS were suspended in 15 µL HBSS and injected in the left myocardium of mouse heart. An analgesic was administrated to mice (subcutaneous injection of 100 µg/kg buprenorphine) for pain relief. Heart was collected 1 month later for immunofluorescence studies.

### Cardiac lymphatic drainage

Six weeks after of TAC or SHAM procedures, mice were anesthetized with isoflurane (2%), 15 µL of 10 mg/mL FITC-2000KDa dextran were injected in the apex of anesthetized mice with a cannula syringe and left to circulate for 10 min before the heart and the caudal mediastinal lymph node were collected. The lymph node was disrupted at 4 °C for 12 h in 0.5% triton, 2% saponin diluted in PBS and FITC dextran present in lymph node was measured with a Tecan spectrofluorometer. The injected hearts were visualized under Macro fluorescence microscope to examine the FITC staining of lymphatic vessels (Leica). In a second approach, 20 µl of 1-μm Fluoresbite green fluorescent plain microspheres diluted 1:2 in sterile PBS were directly injected into cardiac left ventricular wall at one point concomitantly to the realization of TAC. Presence of beads was evaluated 1 week post TAC in cardiac tissue, blood and mediastinal lymph nodes.

### Statistics

All results are presented as mean ± SEM. Two group comparisons were analyzed by unpaired 2-tailed t test. When appropriate, data were analyzed by the 2-tailed Mann–Whitney test. Multiple group comparisons were performed using the 1-way ANOVA followed by Tukey post test. The nonparametric test Kruskal and Wallis followed by Dunn comparison of pairs was used to analyze groups when suitable. Spearman correlation, linear regression and all statistical analysis were performed using GraphPad Prism (version 6.01) software.

## Results

### Expression of LYVE-1 distinguished between two cardiac macrophage subsets

**Figure 1:**
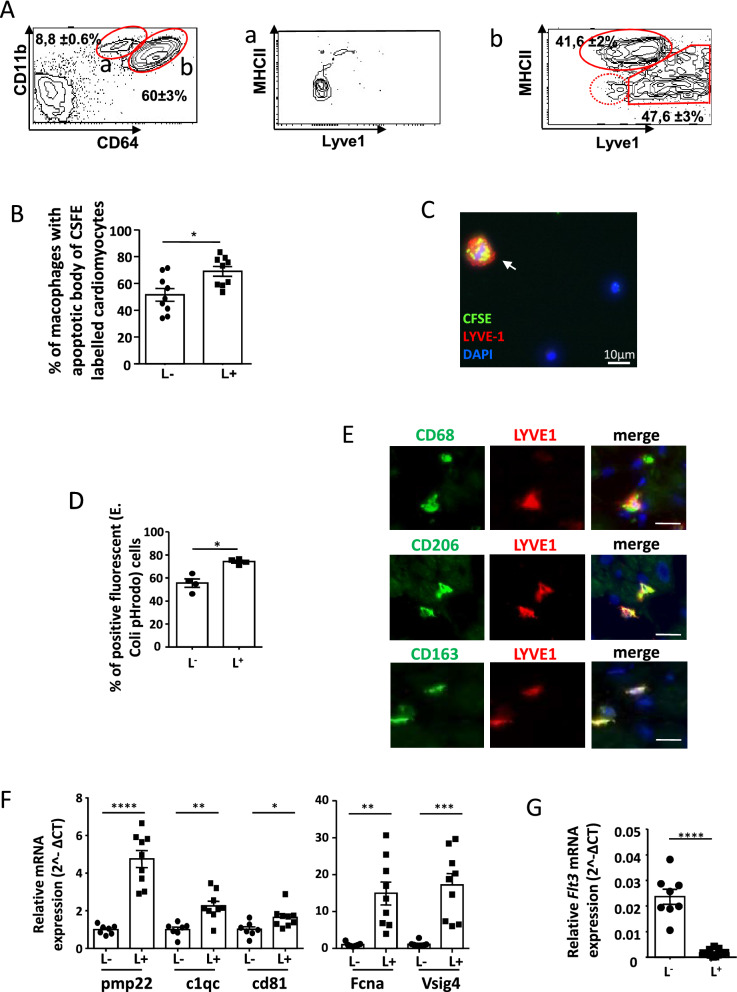
Lyve-1 expression distinguished cardiac macrophage subsets. (**A**) Flow cytometry gating identification of the different monocyte/macrophages populations in cardiac tissue. Cells were first identified on a forward scatter/side scatter (FSC-A/SSC-A) dot plot, the doublets were removed, live cells were selected and CD45 positive and LY6G negative population was analyzed. Then, LYVE-1 and MHCII expression were analyzed in monocytes (CD11b^+^, CD64^low^) and macrophage subsets (CD11b^+^ and CD64^high^ cells). Monocytes (contour plot a) did not express LYVE-1 whereas two main populations of macrophages were divided into LYVE-1 negative (L^−^) and LYVE-1 positive (L^+^) subsets (contour plot b). (**B**) Flow cytometry quantification of efferocytosis in freshly isolated cardiac macrophage subsets showing the percentage of positive cells for CFSE labeled cardiomyocytes. Data are presented in mean ± SEM. *P < 0.05 by *t* test. (**C**) Representative immunofluorescent staining of freshly isolated cardiac CD45 positive cells incubated with CFSE labeled cardiomyocytes, arrow shows LYVE-1 positive cells with abundant phagosomes containing CFSE labeled apoptotic cardiomyocytes. (LYVE-1 was stained in red, DAPI in blue and CFSE in green). Scale bar = 10 µm. (**D**) Quantification by flow cytometry of E. Coli pHrodo phagocytosis in freshly isolated cardiac macrophage subsets. Data are presented in mean ± SEM. *P < 0.05 by Mann–Whitney test. (**E**) Representative images of immunofluorescent staining of LYVE-1 (red) and CD68, CD206 or CD163 (green) in the left ventricular wall of human hearts. Merged images illustrate co-expression and nuclei were stained by DAPI (blue). Scale bar, 20 µm. (**F**) Genes differentially expressed in L− and L+ subsets isolated from mouse hearts. The mean ± SEM corresponds to RT PCR analysis of different isolations of L− and L+ macrophage populations. mRNA expression of gene was normalized to L− subset and p value was determined by t test. (**G**) Expression of Flt3 mRNA expression in L^−^ and L^+^ subsets isolated from mouse cardiac tissue and evaluated by RT-PCR. Data are presented in mean ± SEM. Significance ****P < 0.0001 by *t* test.

Activation of the innate immune response in pressure overload has been associated with impaired ventricular function and heart failure^16,17,32^. We observed that the expression of LYVE-1, a receptor for hyaluronic acid, identified two main cardiac macrophage subpopulations in mouse heart. The subpopulation of macrophages positive for LYVE-1 (L^+^) identified as CD45^+^Ly6G^−^CD11b^+^CD64^hi^L^+^ was mostly MHCII^neg/low^, although a few cells showed a variable level of MHCII as compared with the large population of macrophages negative for LYVE-1 (L^−^) that expressed a high level of MHCII (Fig. 1A). Monocytes identified as CD45^+^Ly6G^−^CD11b^+^CD64^lo^ cells and a small population of CD45^+^Ly6G^−^CD11b^+^CD64^+^ MHCII^neg^ accounting for less than 5% of macrophages, did not express LYVE-1 (Fig. 1A). Immunofluorescence studies confirmed the expression of LYVE-1 in a subset of cardiac macrophages identified by CD68 positive staining (Supplementary Figure 1A). The efferocytic capacity of cardiac macrophages was evaluated by flow cytometry. Freshly isolated L^+^ macrophages exhibited higher ability to engulf apoptotic/necrotic CFSE labelled cardiomyocytes than their L^−^ counterparts and than monocytes (69 ± 3.6% of L^+^ macrophages were CFSE positive vs 51 ± 4.7% of L^−^ macrophages vs 25 ± 2.7% of L^−^MHCII^neg^ macrophages and 19 ± 3.1% for monocytes) (Fig. 1B,C and supplementary Figure 1B,C). Accordingly, the L^+^ macrophages showed a superior capacity to phagocyte E.Coli bioparticles which was consistent with elevated transcription of MerTK and of the scavenger receptors CD206 and CD163 (Fig. 1D, Table 1). In the human heart, the L^+^ macrophages also harbored the scavenger receptors CD206 and CD163 (Fig. 1E). In accordance, the L^+^ subset revealed gene expression associated with resident macrophages (Fig. 1F) while FLT3, a receptor involved in cell survival of hematopoietic stem cells was more expressed in L^−^ macrophages suggesting that higher number of this subset may result from bone marrow-derived monocyte differentiation (Fig. 1G)^33,34^. These data showed that LYVE-1 expression could discriminate peculiar populations of cardiac resident macrophages.

**Table 1:** RT-PCR quantification of CD206, CD163, MERTK, TNF alpha, IL10, Arg1 and Nlrp3 mRNA expression in L^−^ and L^+^ subsets isolated from cardiac tissue after 1 week of TAC or SHAM.

		L−		L+		L+ vs L−
		Mean	SEM	Mean	SEM	
CD163	SHAM	1.02	± 0.06	4.92	0.61	****
	TAC	0.45	± 0.05	4.00	0.50	****
	TAC vs SHAM	###		ns		
CD206	SHAM	1.06	± 0.14	3.10	0.48	**
	TAC	0.68	± 0.07	2.84	0.38	****
	TAC vs SHAM	#		ns		
MERTK	SHAM	1.03	± 0.09	1.74	0.13	***
	TAC	0.80	± 0.05	1.55	0.15	***
	TAC vs SHAM	#		ns		
TNF alpha	SHAM	1.06	0.14	0.65	0.08	*
	TAC	1.55	0.14	0.91	0.10	**
	TAC vs SHAM	#		ns		
NLrp3	SHAM	1.03	0.08	0.75	0.05	*
	TAC	0.99	0.06	0.78	0.05	*
	TAC vs SHAM	ns		ns		
IL1 beta	SHAM	1.00	0.18	0.53	0.08	*
	TAC	0.77	0.10	0.41	0.08	**
	TAC vs SHAM	ns		ns		
Arg1	SHAM	1.15	0.28	2.33	1.14	*
	TAC	1.17	0.30	2.56	0.40	*
	TAC vs SHAM	ns		ns		
IL10	SHAM	1.08	0.14	1.68	0.11	**
	TAC	0.87	0.07	1.39	0.16	**
	TAC vs SHAM	ns		ns		

n = 8 and 9 for L^−^ and L^+^ macrophages respectively in SHAM condition and n = 14 and 11 for L^−^ and L^+^ macrophages respectively in TAC condition. mRNA expression of gene was normalized to housekeeping genes expression and L^−^ subets in SHAM condition. Data are presented in mean ± SEM. Significant differences in mRNA expression was evaluated by Mann–Whitney test between the two subsets (L^+^ vs L^−^) in both SHAM and TAC situations; Significance *P < 0.05; **P < 0.01; ***P < 0.001, ****P < 0.0001 ns indicates not significant, and within the L^−^ and L^+^ subsets during SHAM and TAC (TAC vs SHAM) . Significance ^#^P < 0.05; ^###^P < 0.001, ns indicates not significant.

### The changes in macrophage subsets during chronic pressure overload were associated with the severity of cardiac dysfunction

**Figure 2:**
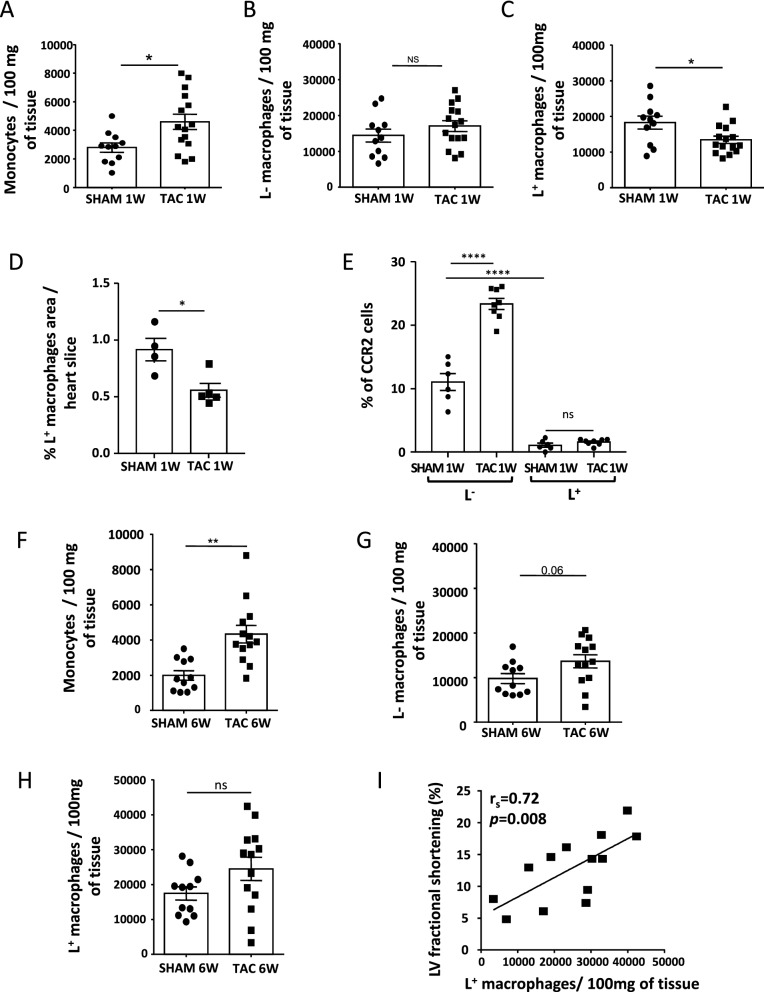
The number of Lyve-1 positive macrophages was inversely correlated with cardiac dysfunction induced by chronic pressure overload. (**A**–**C**) Flow cytometry quantification of monocytes (CD45^+^Ly6G^−^CD11b^+^CD64^lo^) and L^−^ and L^+^ macrophage subsets isolated from cardiac tissue after 1 week of TAC or SHAM. Data are presented in mean ± SEM. Significance *vs* SHAM: *P < 0.05, by *t* test. (**D**) Quantification by immunofluorescent staining of LYVE-1 positive macrophages identified by CD68 in the heart of SHAM mice and after 1 week of TAC. Data are presented in mean ± SEM. *P < 0.05 by Mann–Whitney test. (**E**) Flow cytometric analysis of CCR2 in the two main cardiac macrophage subsets after 1 week of TAC (the percent of CCR2 positive L^−^ macrophages was 11 ± 1% in SHAM vs 23 ± 0.8% in TAC in contrast only 1% of L^+^ subsets were positive for CCR2 in hearts submitted to SHAM or TAC). Data are presented in mean ± SEM. Significance ****P < 0.0001 by ANOVA test. ns indicates not significant. (**F**–**H**) Flow cytometry quantification of monocytes and L^−^ and L^+^ macrophage subsets in cardiac tissue after 6 weeks of TAC or SHAM. Data are presented in mean ± SEM. Significance *vs* SHAM: **P < 0.01, by *t* test. (**I**) Correlation between the number of L^+^ subsets present in cardiac tissue of each individual mouse after 6 week of TAC and the left ventricular fractional shortening (FS). r_s_ = 0.72, p = 0.008 by Spearman. The lines come from a linear regression model.

Next, we investigated the changes in monocyte and macrophage subsets occurring during cardiac remodeling and heart failure induced by pressure overload. One week after TAC, the number of monocytes (CD45^+^CD11b^+^Ly6G^−^CD64^lo^) and CD45^+^CD11b^+^Ly6G^−^CD64^+^ macrophages that did not express L^−^ or MHCII were augmented (Fig. 2A and supplementary Figure 2A). Likewise the main subset of L^−^ macrophages tended to increase in the pathological heart (Fig. 2B). In contrast the L^+^ macrophages slightly decreased after 1 week of TAC (Fig. 2C), their decline was confirmed by immunofluorescence studies (Fig. 2D)*.* Besides the chemokine receptor 2 (CCR2), used to distinguish between macrophages recently derived from circulating monocytes and resident macrophages, was about tenfold more expressed in L^−^ macrophages as compared with L^+^ counterparts and was further increased after TAC in the L^−^ subsets (in SHAM, 12 ± 1.4% of L^−^ macrophages were positive for CCR2 and only 1 ± 0.3% for the L^+^ subset vs 23 ± 0.8% of L^−^ and 1 ± 0.3% of L^+^ in TAC) (Fig. 2E). These observations suggested a high turnover of L^−^ macrophages with a continuous replenishment from inflammatory monocytes during pressure overload. The increase of monocytes and L^−^MHCII^neg^ was still present after 6 weeks of TAC as compared with SHAM (Fig. 2F and supplementary Figure 2C,D) whilst the L^−^ macrophages were faintly augmented (Fig. 2G). In contrast, the number of L^+^ subset found in failing heart was wide ranging and varied according to the cardiac function of the pressure overloaded heart (Fig. 2H,I). These data showed that cardiac hypertrophy and failure induced by pressure overload differently modified macrophage subsets in the heart.

### LYVE-1 positive macrophage subsets displayed a pro lymphangiogenic gene expression profile

**Figure 3:**
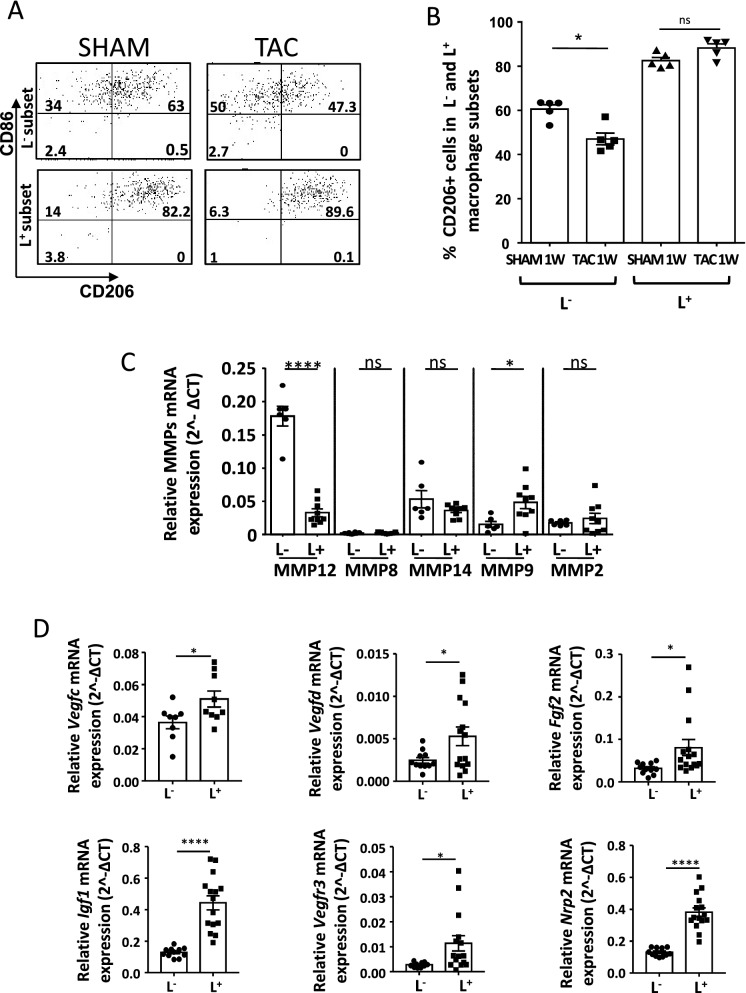
L^−^ and L^+^ macrophage subsets had distinct polarization profiles and expressed pro-lymphangiogenic factors. (**A**,**B**) Flow cytometry dot plots and quantification of CD206 and CD86 expression in L^−^ and L^+^ macrophages present in cardiac tissue after 1 week of TAC or SHAM. Data are presented in mean ± SEM. Significance *P < 0.05; Mann–Whitney test. ns indicates not significant. (**C**) RT-PCR quantification of MMPs expression in L^−^ and L^+^ subsets isolated from SHAM cardiac tissue. mRNA expression of gene was normalized to housekeeping gene expression. Data are presented in mean ± SEM. Significance *P < 0.05; ****P < 0.0001 by Mann–Whitney test. ns indicates not significant. (**D**) RT-PCR quantification of pro-lymphangiogenic growth factors and receptors expression in L^−^ and L^+^ subsets isolated from SHAM cardiac tissue. mRNA expression of gene was normalized to housekeeping genes expression for each group. Data are presented in mean ± SEM. Significance *P < 0.05; ****P < 0.0001 by tests. ns indicates not significant.

To further understand the functions of macrophage subsets, we determined their intrinsic inflammatory status during cardiac remodeling. The two subsets showed different levels of cytokine expression, the L^−^ macrophages expressed more of the pro-inflammatory cytokines TNFα, IL-1β and the inflammasome NLRP3 than L^+^ counterparts which expressed the anti-inflammatory cytokine IL-10 and ARG1 (Table 1). Both L^**+**^ and L^−^ macrophages harbored CD86, a costimulation receptor commonly associated with antigen presentation (Fig. 3A). L^−^ macrophages decreased their surface expression of CD206 after the TAC procedure (60.5 ± 1.9% were CD206^+^ in SHAM *vs* 47 ± 2.6% in TAC) supporting a replenishment with monocytes, whereas CD206 receptors remained unchanged in L^+^ macrophages (Fig. 3A,B). Moreover, the expression of metalloproteinases (MMPs) and of pro-lymphangiogenic factors was different in the two macrophage subsets isolated from both SHAM and TAC hearts. Indeed, the L^−^ macrophages had an abundant expression of MMP12 whereas the L^**+**^ subsets showed higher expression of pro-lymphangiogenic factors IGF1, VEGFc, VEGFd and FGF2 and the receptors VEGFR3 and NRP2 (Fig. 3C,D and data not shown). These data showed that L^−^ and L^**+**^ cardiac macrophage subsets expressed distinct profile of genes and receptors that could support specific functions under cardiac remodeling induced by pressure overload.

### The lymphatic network was remodeled in the pathological heart and the number of LYVE-1 positive macrophages was correlated with the number of lymphatic endothelial cells

**Figure 4:**
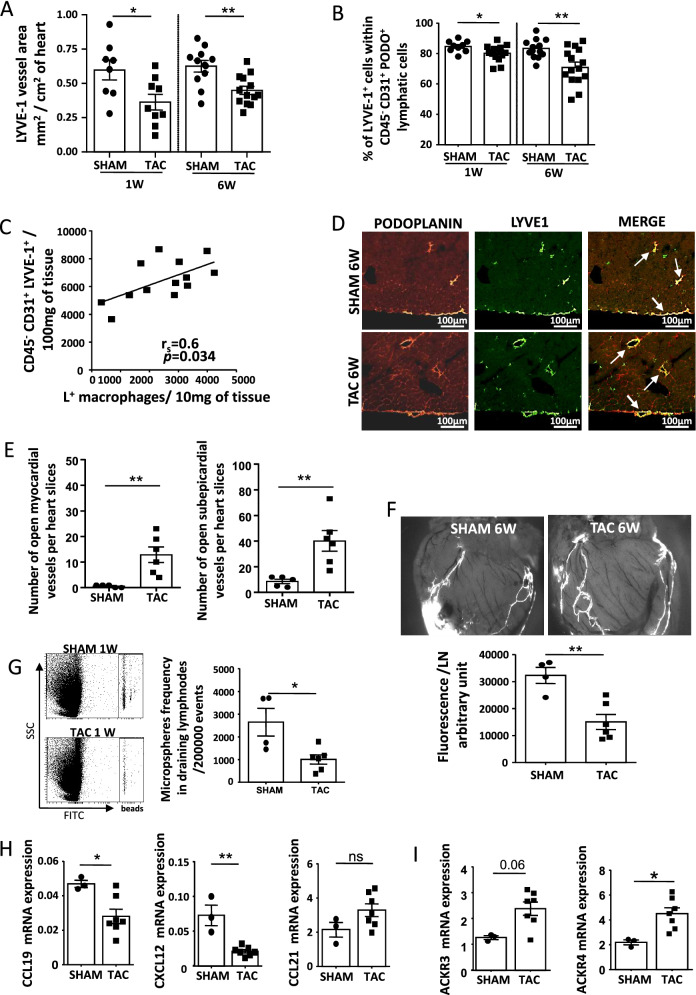
Pressure overload had induced the remodeling of cardiac lymphatic vessels and the numbers of lymphatic endothelial cells was correlated with L^+^ macrophages in hypertrophic and failing hearts. (**A**) Quantification by immunofluorescence of lymphatic vessels found in cardiac tissue after 1 week and 6 weeks of TAC or SHAM. *P < 0.05 **P < 0.01 vs corresponding SHAM, by *t* test. (**B**) Percent of CD45^-^CD31^+^PODO^+^ lymphatic cells expressing LYVE-1 in cardiac tissue after 1 and 6 weeks of SHAM or TAC (flow gating strategy detailed in supplementary 3c). *P < 0.05; **P < 0.01 vs corresponding SHAM by *t* test. (**C**) The number of endothelial lymphatic cells (CD45^-^CD31^+^LYVE-1^+^, present in cardiac tissue of each individual mouse was positively correlated with the number of L^+^ subsets identified after 6 weeks of TAC . r_s_ = 0.6; P = 0.034 by Spearman. The line comes from a linear regression model. (**D**) Representative image of lymphatic vessels in SHAM and chronic failing heart after 6 weeks of TAC (scale bar, 500 µm) and (**E**) quantification of open myocardial and subepicardial lymphatic vessels. Data are presented in mean ± SEM. **P < 0.01 by Mann–Whitney U test. (**F**) Representative staining of lymphatic collectors in heart of mice following 6 weeks of TAC or SHAM, 10 min after the injection of 15 µl of 10 mg/mL FITC—2000 KDa dextran in the apex of heart and quantification of FITC dextran fluorescence in cardiac draining lymph node of mice following injection. Data are presented in mean ± SEM. **P < 0.01; by Mann–Whitney test. (**G**) Representative flow cytometry dot plots of heart draining MLN of mice with SHAM or TAC after intra-myocardial injection of fluorescent beads and their quantification (expressed as number of beads found per 200 000 events) (n = 4–6). *P < 0.05; by Mann–Whitney test. (**H**,**I**) RT-PCR analysis of the CCL19, CXCL12 and CCL21 chemokine expression (**H**) and the atypical chemokine scavenging receptors (ACKR)3 and 4 (**I**) performed on CD45^-^CD31^+^LYVE^−^1^+^ isolated from heart after 1 week of SHAM or TAC. Data were obtained using the comparative 2^−ΔCt^ method to housekeeping gene expression normalization. Data are presented in mean ± SEM of cells isolated from pooled 4 to 6 mouse hearts. Significance *P < 0.05; **P < 0.01 by Mann–Whitney test.

As the cardiac macrophage subsets exhibited differences in lymphangiogenesis associated growth factors and receptors, we examined the lymphatic system during heart remodeling. The heart is provided by a network of lymphatic vessels expressing the endothelial cell marker CD31, VEGFR-3, LYVE-1, and irregularly podoplanin (supplementary Figure 3A,B)^5,35^. In the subepicardium, LYVE-1^+^ vessels coexpressed podoplanin whereas in mid-myocardial areas, podoplanin expression had been not always present in larger size vessels (supplementary Figure 3B). We examined the lymphatic network during cardiac remodeling induced by pressure overload and found that the area of vessels double positive for CD31 and LYVE-1 immunostaining diminished after 1 and 6 weeks of TAC (Fig. 4A). Flow cytometry analysis confirmed a weakened LYVE-1 receptor in cardiac lymphatics during pressure overload as the percentage of CD45^−^CD31^+^PODO^+^ cells expressing LYVE-1 dropped significantly after 1 and 6 weeks of TAC (Fig. 4B and supplementary Figure 3C for gating strategy). The number of CD45^−^CD31^+^LYVE-1^+^ lymphatic cells was inversely correlated with the LV function of chronic pressure overloaded hearts (supplementary Figure 4C). The decrease of lymphatic vessels was attested by a lessening of the cardiac superficial epicardial LYVE-1 positive lymphatic network (supplementary Figure 4A) and a reduction of total CD45^−^CD31^+^LYVE-1^+^ cells in heart after 1 week of TAC (supplementary Figure 4B). After 6 weeks of TAC, the number of CD45^−^CD31^+^LYVE-1^+^ lymphatic cells found in the heart was positively correlated with the number of L^+^ macrophages showing a tight regulation of the lymphatic network during heart failure (Fig. 4C). Moreover, the failing hearts exhibited a higher number of lymphatic vessels with open lobular lumens (Fig. 4D,E). To determine whether lymphatic vessel modifications reshaped communication between the heart and draining lymph nodes we evaluated the capacity of heart to draw off FITC-dextran injected in apex. The mediastinal draining lymph nodes (MLNs) of the failing heart exacerbated a reduced fluorescence compared with SHAM mice (Fig. 4F). In a second approach we analyzed, in the MLNs, the presence of fluorescent beads directly injected into cardiac tissue concomitantly to TAC. The number of beads that had reached the MLNs was significantly reduced after TAC, confirming a dysfunctional lymphatic drainage during pressure overload (Fig. 4G). The endothelial receptor LYVE-1 serves as a docking to leukocyte entry into the lymphatic; hence we examined the chemokines involved in recruitment of immune cells. Expression of CCL19 and CXCL12 was reduced in the CD45^−^CD31^+^LYVE-1^+^ cells isolated from TAC mice while ACKR3 and 4 (atypical chemokine receptors), the scavenging receptors for CXCL12 and CCL19 respectively, were up regulated (Fig. 4H,I). These results showed that pressure overload remodeled the lymphatic network, altered the drainage and the regulation of chemokines and receptor expression in lymphatic cells. In addition, our data highlighted an unknown correlation between the CD45^−^CD31^+^LYVE-1^+^ lymphatic cells and a subset of cardiac macrophages.

### LYVE-1 positive macrophages exhibited pro-lymphangiogenic functions

**Figure 5:**
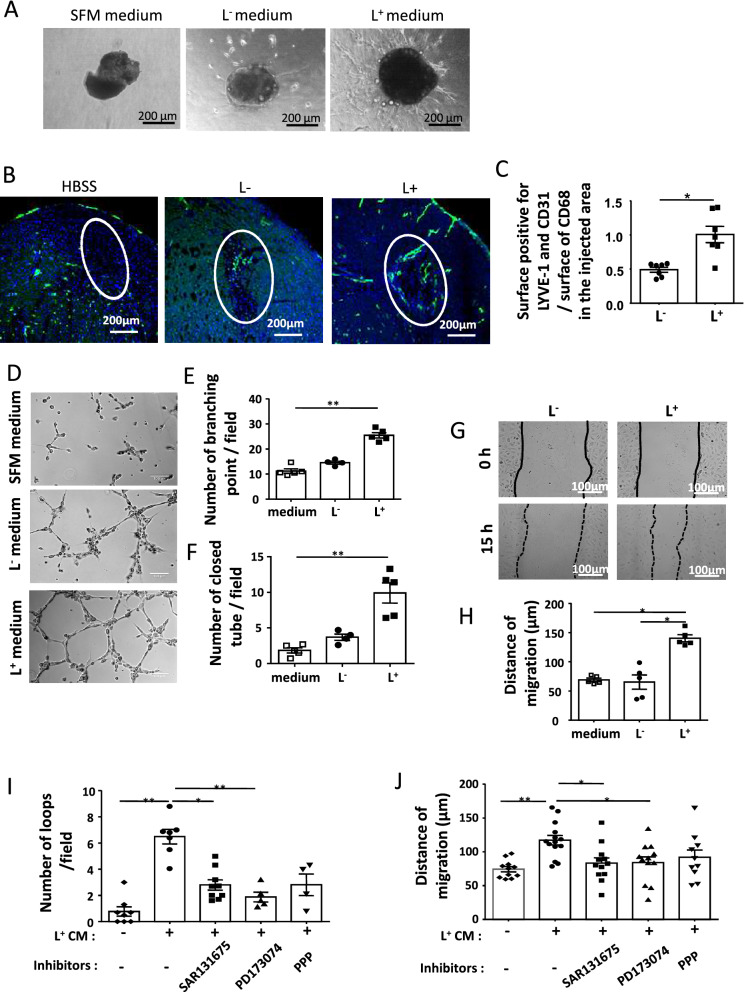
L^+ ^macrophages expressed in vitro pro-lymphangiogenic activity. (**A**) Representative images of lymphatic duct explants embedded in type I collagen gel and cultured for 10 days in conditioned medium of cardiac isolated L^−^ or L^+^ macrophages or control media (SFM). 3 independent experiments. (**B**) LYVE-1 immunofluorescent staining of hearts injected with HBSS alone, or with 200,000 L^−^ or L^+^ macrophages. Dotted circles indicate the injected area identified by a high density of cell nuclei and a disorganized tissue showing the presence of nascent Lyve-1 positive structures. (**C**) Quantification of CD31^+^ LYVE-1^+^ structures normalized to the amount of CD68 found in the injected area. HBSS injected hearts did not exhibited LYVE-1 positive structure in the injected area. Data are presented as mean of 3 to 4 slices of injected hearts ± SEM. *P < 0.05; by *t* test. (**D**) Representative images of capillary-like structures formed by HDLECs when plated on growth factor-reduced Matrigel-coated tissue culture plates and incubated with conditioned medium of cardiac L^−^ or L^+^ macrophages or SFM (medium) diluted at ¼ in EGM2 without growth factors supplementation. (**E**) The number of branching points and (**F**) closed tube-like (loop) structures were quantified in 6 high power fields. (**P < 0.01 by Kruskal–Wallis test). (**G**) Representative images of wound healing assay to assess the migration of HDLECs and incubated with conditioned medium of cardiac L^−^ or L^+^ macrophages or SFM (medium) diluted at ¼ in EGM2 without growth factors supplementation. Black dotted lines outline wound area at indicated time points. (**H**) Total distance of migration was measured by the difference between the two time points and reported. Data are presented in mean ± SEM and are representative of 3 independent experiments. Significance *P < 0.05; by Kruskal–Wallis test. (**I**) Quantification of capillary-like structures formed by HDLECs plated on growth factor-reduced Matrigel and incubated with conditioned medium of lymphangioma isolated L^+^ macrophages in presence of the different inhibitors at the concentration of 100 nM for SAR131675 and PD173074 or 0.5 µM of PPP. The number of closed tube-like (loop) structures was quantified after 4 h in high power fields. (**J**) Quantification of migration of HDLECs evaluated with wound healing assay and incubated with conditioned medium of lymphangioma isolated L^+^ macrophages in presence of the different inhibitors at the concentration of 100 nM for SAR131675 or PD173074 and 0.5 µM for PPP. Significance *P < 0.05; **P < 0.01; by one way ANOVA.

To further address the interplay between macrophage subsets and lymphatic vessels, we analyzed the macrophage populations in a model of induced lymphangiogenesis. The lymphangioma was produced by the injection of incomplete Freund's adjuvant, first developed by Jamison et al. and used by different groups^36,37^. Thus, we observed that 10 to 20% of macrophages identified as CD45^+^CD11b^+^ and CD64^+^ cells were also positive for LYVE-1 (supplementary Figure 5A). As observed in the heart, the L^**+**^ macrophages isolated from lymphangioma expressed significantly more the pro-lymphangiogenic factors and the genes associated with resident macrophages than their corresponding L^−^ subsets (supplementary Figure 5B). To establish the pro-lymphangiogenic potential of the macrophage subsets, we used a three-dimensional culture system of lymphatic ring developed by Bruyere et al.^37^. Murine thoracic duct fragments embedded into type I collagen gels showed an important lymphatic sprouting when cultured in conditioned media (CM) of L^**+**^ macrophages isolated from both cardiac and lymphangiomas as compared with CM of L^−^ counterparts. The control medium showed no outgrowth or few cells surrounding the vessel explants indicating that macrophage media contained sufficient factors to allow the formation of vessel outgrowths (Fig. 5A and supplementary Figure 5C). To further examine the potency of L^+^ macrophages to promote lymphangiogenesis, we injected into the left wall either 200,000 L^+^ or L^−^ cells isolated from lymphangioma in HBSS solution. One month later, assembled structures expressing LYVE-1, the endothelial cell marker CD31 and discontinuous level of podoplanin were abundantly present in the injection area of L^+^ macrophages as compared with area injected with L^−^ macrophages (Fig. 5B,C, supplementary Figure 5D). No LYVE-1 positive staining was observed in HBSS injected heart (Fig. 5B). Interestingly, the existence of L^+^ macrophages in the lining of the nascent lymphatic-like structures was revealed by double staining with the myeloid cell marker CD11b and with the macrophage marker CD68 (supplementary Figure 6A–C). The occurrence of CD68 positive cells into cardiac lymphatic capillaries was also frequently observed in control heart (data not shown). To decipher the mechanisms involved, we examined the formation of capillary-like structures of primary human dermal lymphatic endothelial cells (HDLECs) plated on growth factor reduced Matrigel. The presence of cardiac and lymphangioma isolated L^+^ macrophage conditioned media (CM) was more effective in promoting tube formation (Fig. 5D–F) and to enhance the ability of HDLECs to migrate as revealed by the scratch tests, than L^−^ macrophage CM and control medium (Fig. 5G,H, supplementary Figure 6D,E). The use of different receptor inhibitors showed that different pro lymphangiogenic growth factors contributed to the effects of L^+^ macrophage conditioned media. Indeed, the VEGFR3 inhibitor SAR131675 and the FGFR1 inhibitor PD173074 significantly inhibited both HDLEC branching and migration, whereas picropodophyllin, an IGF-1R inhibitor induced a lower and non significant reduction (Fig. 5I,J) revealing the participation of both VEGFC-D and FGF2 in these processes.

### LYVE-1 negative macrophages were able to shed the endothelial LYVE-1

**Figure 6:**
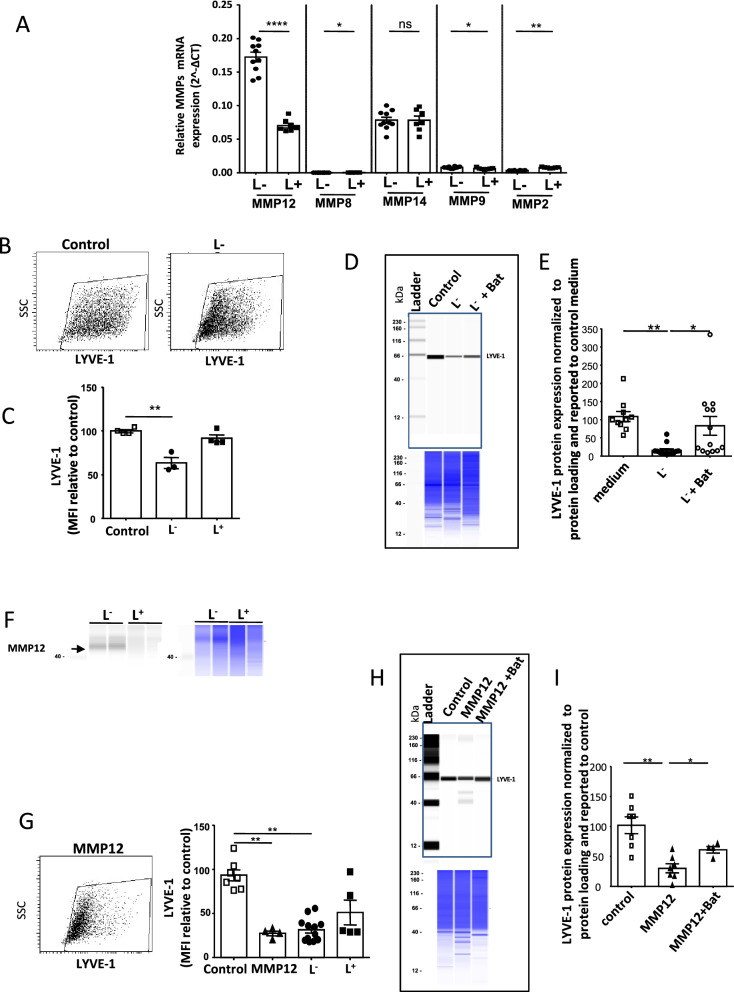
Metalloprotease activity of L^−^ macrophages promoted LYVE-1 shedding. (**A**) RT-PCR quantification of MMPs expression in L^−^ and L^+^ subsets isolated from lymphangioma. mRNA expression of gene was normalized to housekeeping gene expression for each group. Data are presented in mean ± SEM. Significance *P < 0.05;; **P < 0.01; ****P < 0.0001 by Mann–Whitney test. ns indicates not significant. (**B**) Representative flow cytometry staining of control LYVE-1-transfected CHO cells (left panel) after 12 h incubation with conditioned medium of L^−^ macrophages isolated from heart (right panel). (**C**) Quantification of LYVE-1 level (MFI) on live CHO transfected cells incubated with conditioned medium of L^−^ or L^+^ macrophages isolated from heart. Data are presented in mean ± SEM. Significance **P < 0.01 vs control incubated with p-aminophenylmercuric acetate by Mann–Whitney test. (**D**) Representative jess immunodetection on cell lysates obtained from HLEC incubated with conditioned media from lymphangioma isolated L^−^ macrophages for 36 h in absence or not of 10 µM Batimastat. Lower panel shows corresponding total protein load. (**E**) Quantification of LYVE-1 in HLEC following incubation with L^−^ macrophage conditioned media. Data are presented in mean ± SEM. Significance *P < 0.05; **P < 0.01 by Kruskal–Wallis test. (**F**) Representative immunodetection of MMP12 in lymphangioma isolated L^−^ and L^+^ macrophages (left panel) and quantification of loaded proteins (right panel). (**G**) Representative flow cytometry staining of LYVE-1-transfected CHO cells incubated for 12 h with 400 ng of recombinant MMP-12 activated with p-aminophenylmercuric acetate and quantification of LYVE-1 MFI on live transfected CHO cells after 12 h incubation with MMP12 or with conditioned medium of L^−^ or L^+^ macrophages isolated from lymphangioma (right panel). Data are presented in mean ± SEM. Significance **P < 0.01 vs control incubated with p-aminophenylmercuric acetate by Mann–Whitney test. (**H**,**I**) Representative jess immunodetection on cell lysates obtained from HLEC incubated with 500 ng of recombinant MMP12 protein for 5 h and preincubated or not of 10 µM Batimastat as described in supplementary methods and quantification (**I**). Data are presented in mean ± SEM. Significance *P < 0.05; **P < 0.01 by Kruskal–Wallis test.

The cell surface expression of endothelial LYVE-1 was blunted during pressure overload and LYVE-1 receptor presents numerous cleavage sites targeted notably by the proteinases MMP14 and ADAM17. The profile of proteinase expression was different in the L^−^ and L^+^ macrophage subsets isolated from both cardiac and lymphangioma (Figs. 3C, 6A), thus we examined the effect of macrophage conditioned media (CM) on LYVE-1 protein. For this purpose, we transfected CHO cells to express LYVE-1, then the receptor present on cell surface was analyzed by flow cytometry. The incubation with L^−^ macrophage CM significantly reduced LYVE-1 staining (Fig. 6B,C). A similar reduction of LYVE-1 protein expressed by HLEC was observed with L^−^ CM and was abolished by the presence of the MMP inhibitor batimastat (Fig. 6D,E).

Gene expression analysis showed a robust expression of MMP12 in this subset that was confirmed by western blotting (Fig. 6F, full size capillaries available in supplementary Figure 6D). Thus we examined the effect of purified MMP12 on LYVE-1 protein. MMP12 triggered a loss of LYVE-1 detection both on LYVE-1 transfected CHO cells (Fig. 6G) and HLEC that was prevented by batimastat (Fig. 6H,I). These data indicated that the L^−^ macrophage subset was able to regulate LYVE-1 shedding a feature of lymphatic cells associated to pressure overload and provided evidence for distinct functions associated with macrophage subsets.

### The inhibition of CCR2^+^ monocyte infiltration modified cardiac macrophage subsets and prevented lymphatic remodeling after TAC

**Figure 7:**
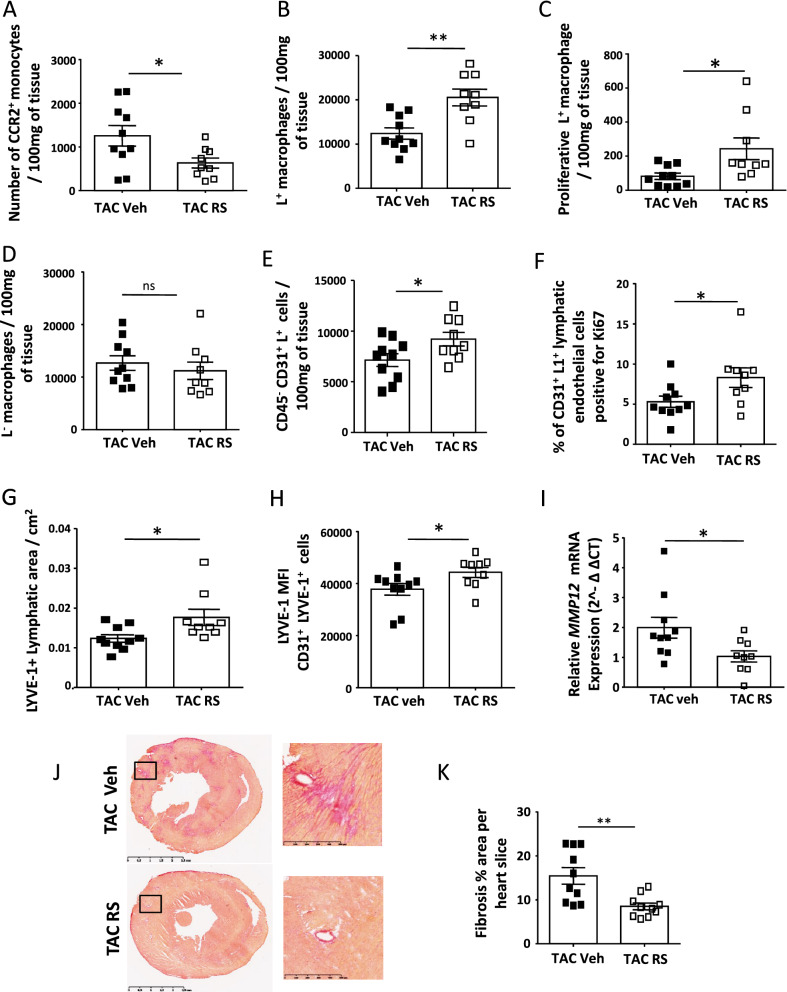
The inhibition of CCR2 positive monocyte infiltration changed the cardiac proportion of L^−^ and L^+^ macrophage subsets and modified the lymphatic network. (**A**) Number of monocytes positive for CCR2 after one week of TAC in heart of mice treated with either vehicle (TAC veh) or RS504393 (TAC RS) *P < 0.05 by *t* test. (**B**) Quantification of L^+^ macrophages present in heart of vehicle (TAC veh) and RS504393 (TAC RS) treated mice. **P < 0.01 by *t* test. (**C**) Flow cytometry quantification of Ki67 positive L^+^ macrophages in cardiac tissue. *P < 0.05 by *t* test. (**D**) Flow cytometry quantification of L^−^ macrophages present in heart of vehicle (TAC veh) and RS504393 (TAC RS) treated mice. (**E**) Quantification of lymphatic endothelial cells by flow cytometry identified as CD45^−^CD31^+^LYVE-1^+^ in cardiac tissue of TAC vehicle and TAC RS504393. *P < 0.05 by *t* test (Gating strategy detailed in supplementary Figure 3C). (**F**) Percentage of Ki67 positive lymphatic endothelial cells identified and evaluated by flow cytometry in cardiac tissue of TAC vehicle and TAC RS504393. (**G**) Immunofluorescence quantification of lymphatic vessel area in cardiac tissue of mice submitted to 1 week of TAC and treated with either vehicle (TAC veh) or RS504393 (TAC RS). *P < 0.05; by *t* test. (**H**) Flow cytometry determination of LYVE-1 expression (mean fluorescence intensity or MFI) on CD45^-^CD31^+^LYVE-1^+^ cardiac endothelial lymphatic cell surface after 1 week of TAC of mice treated with either vehicle (TAC veh) or RS504393 (TAC RS). Data are presented in mean ± SEM. Significance *P < 0.05; by *t* test. (**I**) Relative expression of MMP12 in heart of mice submitted to 1 week of TAC and treated with either vehicle (TAC veh) or RS504393 (TAC RS). mRNA expression of gene was normalized to TAC RS, *P < 0.05; by *t* test. (**J**,**K**) Representative images of Sirius red staining of heart after 1 week of TAC from mice treated with either vehicle (TAC veh) or RS504393 (TAC RS) and quantification of fibrosis content (**K**) . **P < 0.01; by *t* test.

To explore the influence of L^−^ to L^+^ macrophage subsets ratio in lymphatic remodeling during TAC, we treated mice with the CCR2 chemokine receptor antagonist RS504393 to limit the turnover of monocyte-derived L^−^ macrophages during pressure overload. As expected RS504393 treatment induced an abrogation of CCR2^+^ monocytes rise after 1 week of TAC consistent with a previous report (Fig. 7A) and modified the proportion of macrophage subsets in hypertrophic hearts^38^. Indeed, the RS504393 treatment rose significantly the L^**+**^ macrophages (12,415 ± 1293 L^+^/100 mg of heart in TAC vehicle vs 20,557 ± 1885 in TAC RS504393) through an increase in their proliferating rate (75 ± 19 CD45^+^CD11b^+^CD64^hi^LYVE-1^+^/100 mg of heart were Ki67^**+**^ in TAC vehicle *vs* 240 ± 65 in TAC treated with RS504393) (Fig. 7B,C) while the cardiac population of L^−^ MHCII^neg^ decreased (supplementary Figure 7A) and the L^−^ subset tended to diminish (12,660 ± 1381 L^−^/100 mg of heart in TAC vehicle *vs* 11,210 ± 1653 in TAC treated with RS504393*)* (Fig. 7D)*.* Thus RS504393 treatment restored, after 1 week of TAC, a ratio between the L^+^ subset and the L^−^ subset similar to SHAM mice. In addition the RS504393 treated hearts exhibited a higher number of lymphatic endothelial cells (6850 ± 595 CD45^−^CD31^+^LYVE-1^+^/100 mg heart of TAC vehicle *vs* 9214 ± 668 in TAC RS504393) (Fig. 7E) through an increase in cell proliferation rate (Fig. 7F) and a larger lymphatic vessels surface positive for LYVE-1 than in vehicle treated hearts (Fig. 7G). The treatment of mice with the RS504393 preserved the expression of LYVE-1 protein on cardiac endothelial cells (Fig. 7H, supplementary Figure 7B) along with a significant reduction of cardiac MMP12 expression after 1 week of TAC (Fig. 7I). Furthermore, the ejection fraction was significantly preserved in CCR2 antagonist treated mice (Table 2) and the reduced pathological remodeling was confirmed by cardiac gene expression and fibrosis analysis in RS504393 treated hearts (supplementary Figure 7C,D, Fig. 7J,K). To further explore the participation of newly recruited bone marrow-derived macrophages in hypertrophic hearts, we examined chimeric mouse hearts in which CD45.1 bone-marrow was transferred into irradiated CD45.2 recipient mice after 1 week of TAC. Immunofluorescence studies showed that infiltrated CD45.1 cells did not express LYVE-1 and were essentially concentrated in foci of interstitial collagen III enriched areas (supplementary Figure 7E). Indeed, myocardial areas of fibrosis after TAC did not contain LYVE-1 macrophages or lymphatic vessels, although the abundant presence of macrophages (supplementary Figure 7F). These data emphasized that cardiac macrophage subsets supported different functions in lymphatic remodeling and showed that modulation of these populations of macrophage during pressure overload interfered with lymphatic network remodeling.

**Table 2:** Echocardiographic parameters of WT mice treated with either vehicle or RS504393 after 1 week of transverse aortic constriction (TAC).

	TAC Veh	TAC RS
*n*	11	10
Heart rate (bpm)	455 ± 43	421 ± 15
LVIDs (mm)	3.6 ± 0.12	3.13 ± 0.15**
LVIDd (mm)	4.25 ± 0.09	4.04 ± 0.12
Ejection fraction (%)	32.64 ± 2.94	45.78 ± 3.58**
FS (%)	15.52 ± 1.55	23.13 ± 1.98**
LVAW;s (mm)	1.27 ± 0.07	1.21 ± 0.04
LVAW;d (mm)	1.02 ± 0.05	0.89 ± 0.03
LVPW;s (mm)	1.04 ± 0.04	1.08 ± 0.03
LVPW;d (mm)	0.87 ± 0.04	0.81 ± 0.04

LVAW;d and LVAW;s indicate left ventricle anterior wall thickness at end diastole (d) or end systole (s) respectively; LVPWd and LVPWs end diastolic and end systolic left posterior wall thickness respectively; LVIDd, diastolic left ventricular dimension; LVIDs, systolic left ventricular dimension and FS, fractional shortening (%). Values shown are mean ± SEM. Significance vs TAC vehicle: **P < 0.01 by *t* test.

## Discussion

The morphology of cardiac lymphatic was described in early twentieth century, but the evolution of this network during cardiac pathologies is poorly defined^39^. Chronic ventricular pressure overload produced an enlargement of lymphatic vessels, a decline in lymphatic efficiency along with a global loss of LYVE-1 receptor in cardiac lymphatic vessels. The increase of the open vessels ratio found in mouse heart after TAC conforms to the pattern previously reported for initial lymphatics of patients with terminal heart failure^40^. Precollectors of rodent hearts are devoid of smooth muscle cells described to generate lymph motion cycles of contraction and relaxation^41^. Therefore ventricular contraction strength altered in heart failure could, in part, explain the lower efficiency of heart to draw off injected FITC dextran to mediastinal lymph after 6 weeks of TAC. However, a reduced number of fluorescent beads reached draining lymph nodes during the first week of TAC along with a decline of key chemotactic factors in cardiac lymphatic cells. Indeed, expression of CXCL12 and CCL19 chemokines was down regulated whereas the atypical chemokine receptors (ACKRs) 3 and 4 were up-regulated in lymphatics during pressure overload. ACKR4 is an atypical receptor for CCL19 and CCL21 and ACKR3 binds CXCL12, both receptors fail to transduce signals and consequently cellular responses, but instead internalize and scavenge the chemokines to lower their availability. The down-regulation of LYVE-1 receptor expressed by endothelial cells may also contribute to modify the lymphatic ability to recruit and transport immune cells to draining lymph nodes during pressure overload. Indeed, lymphatic endothelial LYVE-1 was described to serve as a docking receptor for hyaluronic acid-coated leukocytes and the endothelial deletion of LYVE-1 promoted the accumulation of macrophage in the ischemic heart^4,25^. The loss of signals that drive leukocyte trafficking associated with a reduced lymphatic drainage appeared as mechanisms settled to preserve the draining lymph nodes from the recruitment of self-antigen loaded presenting cells during pressure overload and thus, may limit immune responses. The loss of LYVE-1 in lymphatic cells observed during pressure overload appeared regulated by cardiac macrophage populations. Indeed, the pattern of MMPs specific to the L^−^ macrophages endowed capacity to reduce LYVE-1 staining on both lymphatic endothelial cells and CHO cells expressing LYVE-1. The shedding of the LYVE-1 receptor could be reproduced by purified MMP12, an MMP highly associated with inflammatory diseases and described to promote the proteolysis of several extracellular matrix components, cytokines and chemokines^42–44^. However, numerous cleavage sites targeted by the proteinases MMP14 and ADAM17 have been identified in the juxtamembrane location of LYVE-1 receptor, therefore additional proteinases might also have contributed to the cleavage of LYVE-1 by L^−^ macrophage conditioned media^24,25^.

A peculiar macrophage population that differentiated from their counterpart by their low expression of CCR2 and Flt3 and by resident macrophage gene expression signature was associated with preservation of the lymphatic network during cardiac remodeling. The number of this macrophage subset varied accordingly to the number of lymphatic cells within the pathological hearts, suggesting a tight interplay between these two cell populations. This L^+^ macrophage subset, also present in an induced model of lymphangiogenesis, expressed the growth factors VEGFC-D, FGF2 and IGF-1. In contrast to newly recruited bone marrow-derived macrophages that accumulated essentially in fibrotic areas of hypertrophic hearts, the L^+^ macrophages were recurrently present into the lining of neo-formed or existing cardiac lymphatic capillaries. In the brain, macrophages were described as cellular chaperones for vascular anastomosis of nascent sprouts^45^. They also participate to control arterial stiffness and structure by regulating collagen^28^. In the heart, we found that L^+^ macrophages renew by in situ proliferation and were able to stimulate pro-lymphangiogenesis both in vivo*, *ex vivo and in vitro*,* by mechanisms dependant of pro-lymphangiogenic growth factors VEGFC-D and FGF2. The contribution of macrophage subsets in lymphatic network remodeling during pressure overload was further supported by pharmacological treatment with CCR2 antagonist. The reduction of CCR2 dependent monocyte recruitment during TAC dampened MMP-12 expression, abrogated the loss of LYVE-1 on lymphatic cells and maintained L^+^ macrophages by enhancing their proliferation. Little is known about factors that increase cell cycle entry and sustain in situ proliferation of macrophages. Pro-inflammatory cytokines such as TNF-α, have been described to inhibit proliferation of adipose tissue macrophages^46^. As CCR2 L^−^ macrophages appeared to express pro- inflammatory factors, they might have locally restrained L^+^ macrophage self renewal. The close localization of L^+^ macrophages to cardiac lymphatic capillary structure coupled to their ability to stimulate pro-lymphangiogenesis in vivo and ex vivo argued for their regulatory role on lymphatic vessels under pathological conditions. In addition, L^+^ cardiac macrophages may also promote cardioprotective functions through anti inflammatory and pro resolutive proprieties, essential to preserve the heart from maladaptive ventricular remodeling^47^.

Nevertheless a distinct contribution of macrophage subsets to the outcome of lymphatic remodeling was provided by CCR2 antagonist treatment that preserved cardiac macrophage subsets ratio and reduced lymphatic remodeling within the hypertrophic heart. Our study showed regulation of the cardiac lymphatic vessels upon pressure overload and revealed novel and unexpected functional role of macrophage subsets in lymphatic remodelling during pressure overload.

## Supplementary Information

Supplementary Information.

Supplementary Figures.

## Abbreviations

LYVE-1: Lymphatic vessel endothelial hyaluronic acid receptor-1
L^−^ and L^+^: LYVE-1 negative and positive cells respectively
TAC: Transverse aortic constriction
CM: Conditioned media
