# Supplementary material for: Cardiac macrophage subsets differentially regulate lymphatic network remodeling during pressure overload
Source: Sci Rep. 2021 Aug 19;11:16801. doi: 10.1038/s41598-021-95723-y (PMC8376913; doi:10.1038/s41598-021-95723-y)

Supplementary Figure 1

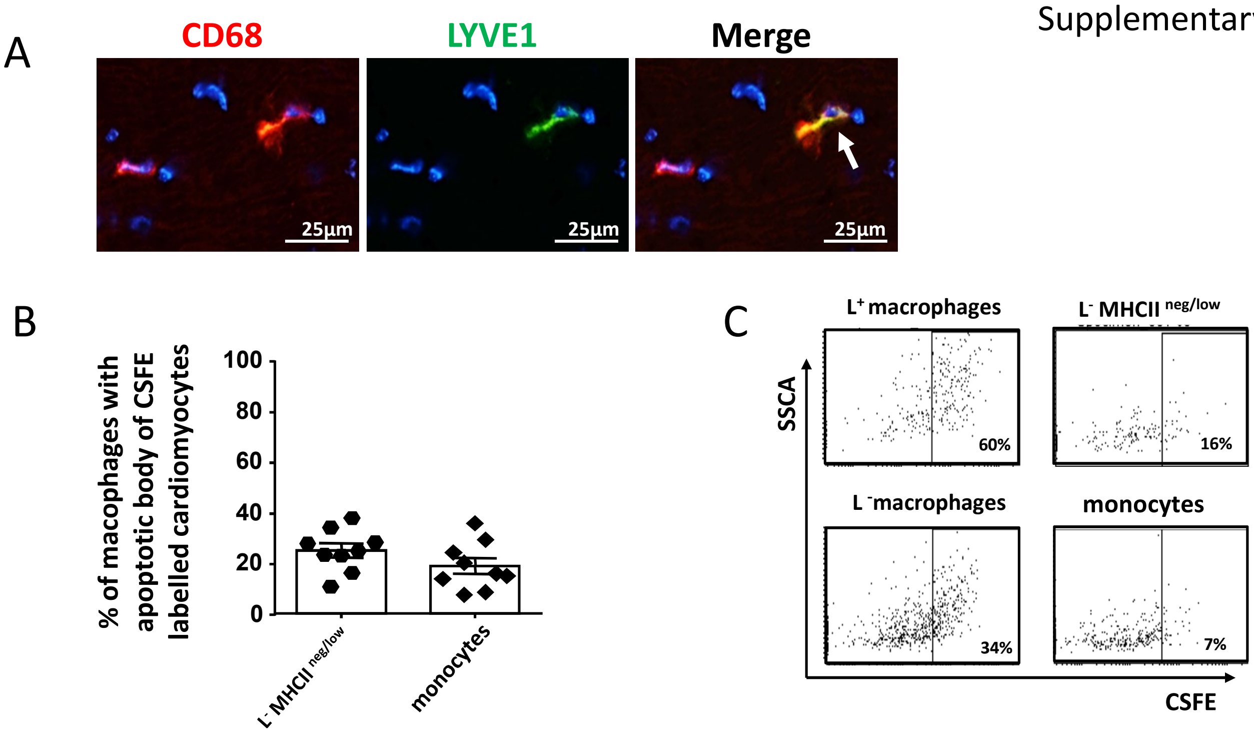

Supplementary Figure 2

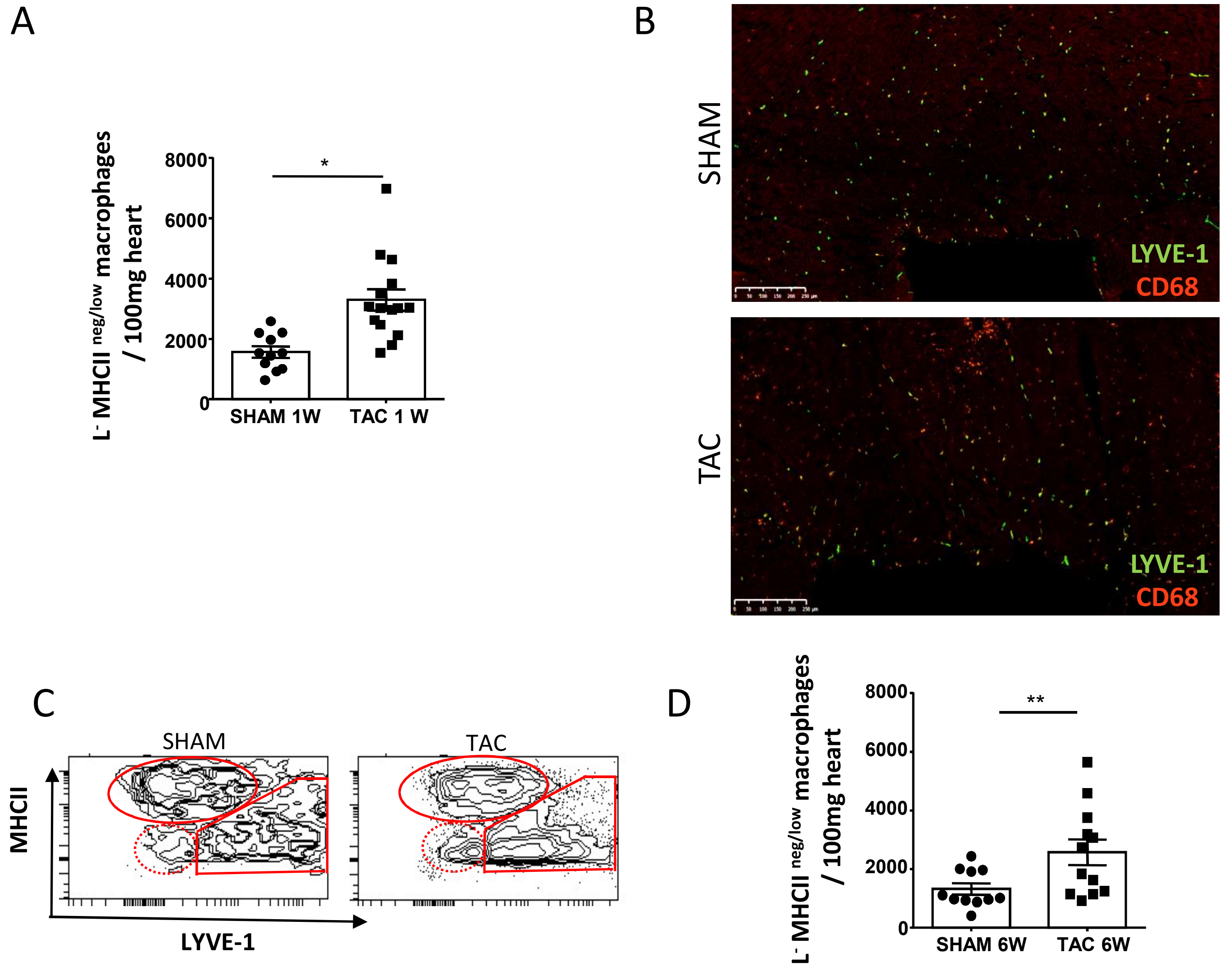

Supplementary Figure 3

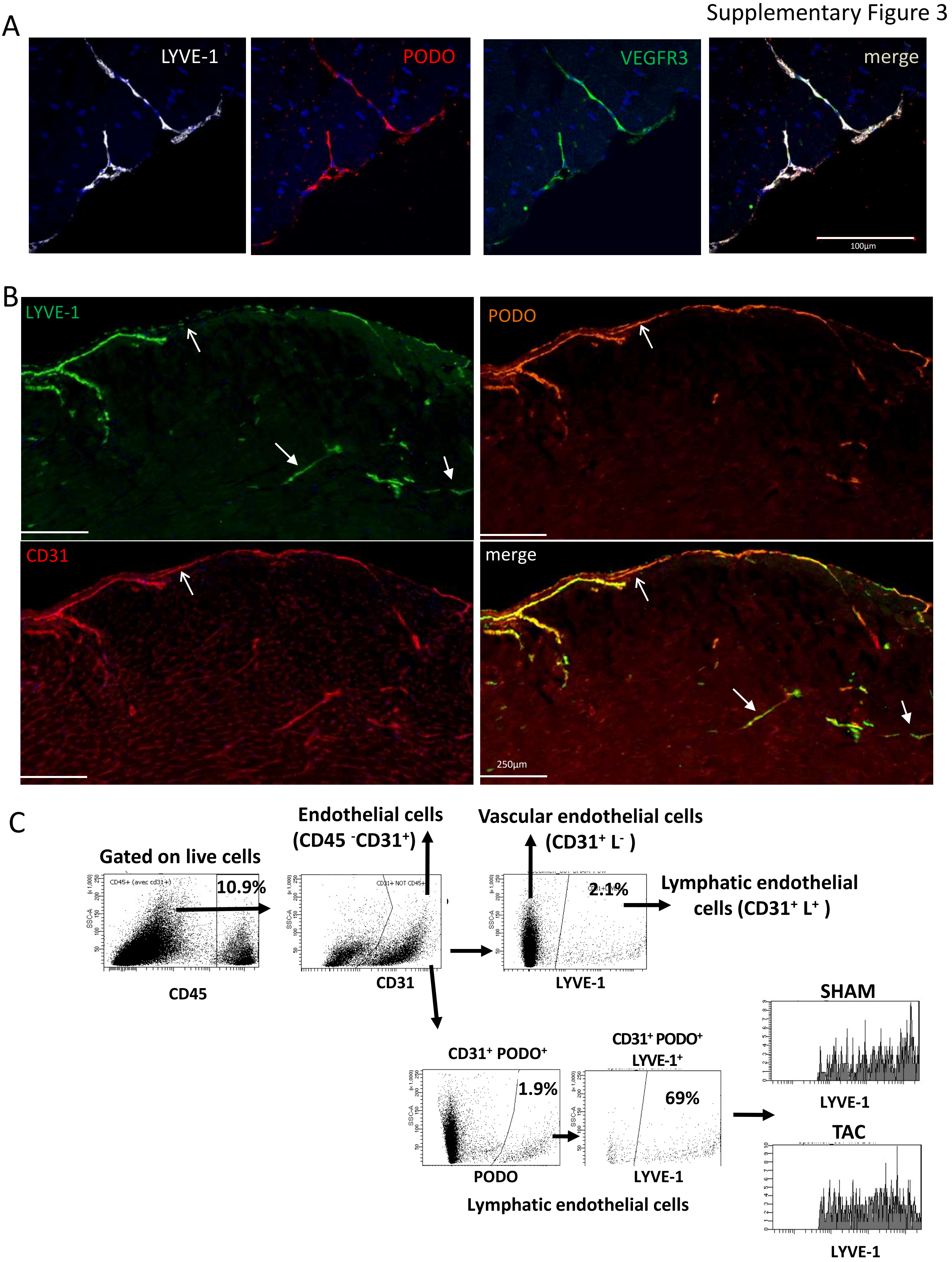

Supplementary Figure 4

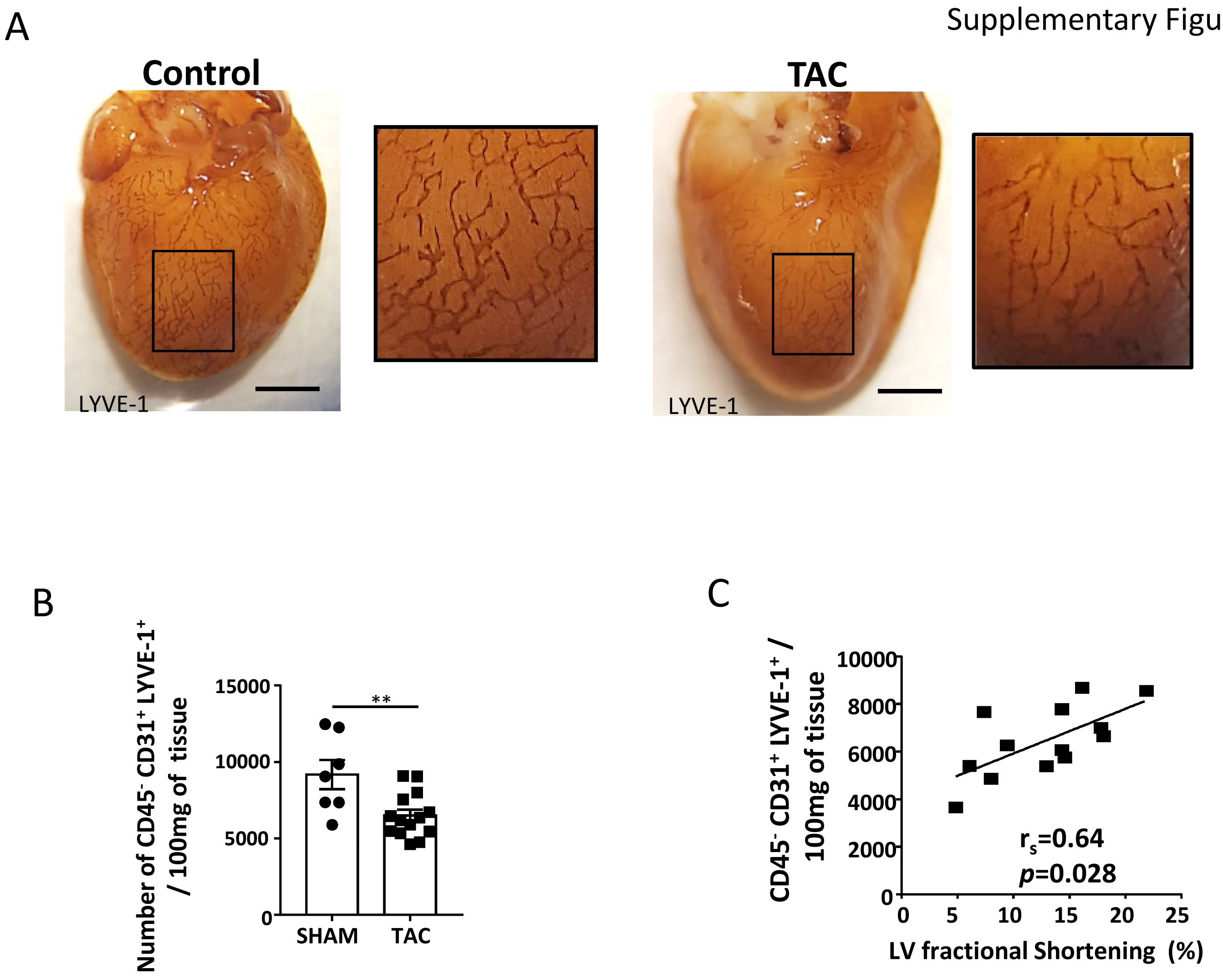

Supplemental Figure 5

A

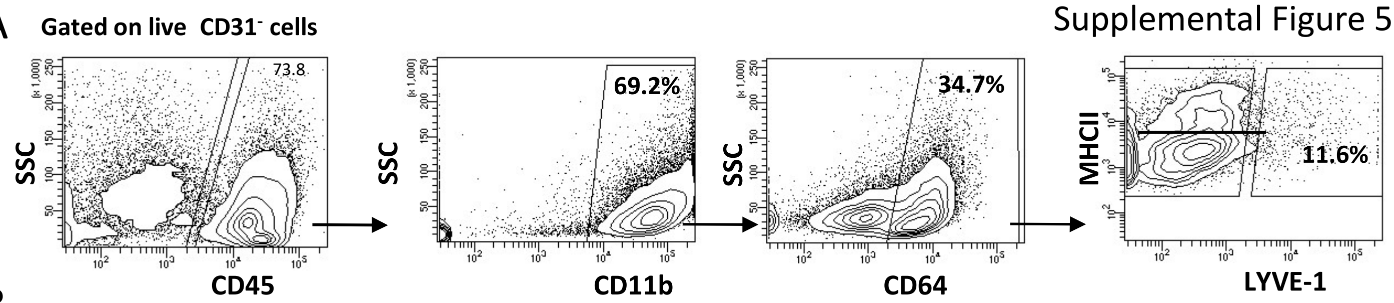

B

	L-					Mean ± SEM	L+					Mean ± SEM	*p value*
LYVE1	0,6	1,1	0,4	1,0	1,0	1,25±0,5	132,1	65,9	48,2	168,5	133,3	90,5±18	0,001
VEGFR3	0,5	1,0	0,7	0,7	1,4	1,18±0,3	10,8	7,9	12,9	64,5	66,5	20,5±7,5	0,030
IGF1	1,0	0,6	0,6	1,0	1,0	1,17±0,3	2,3	1,8	3,0	1,7	1,3	2,1±0,3	0,048
FGF2	1,0	0,8	1,0	0,9	1,1	1,07±0,3	4,9	4,8	4,1	2,9	2,0	3,2±0,5	0,009
VEGFD	0,8	1,5	0,8	0,7	1,5	1,24±0,3	2,0	3,1	2,4	4,4	5,0	2,6±0,4	0,027
VEGFC	0,7	0,9	1,1	1,4	0,7	1,19±0,4	2,2	1,6	1,7	4,3	3,3	3,3±0,9	0,04
CD206	1,5	1,6	1,0	1,2	0,7	1,11±0,2	12,0	11,9	21,8	18,7	17,9	14,9±5,3	0,05
CD163	0,5	0,8	1,1	0,7	0,8	1,16±0,3	21,1	14,5	20,4	16,7	8,6	14,8±3,3	0,007
MERTK	1,1	0,9	0,8	0,9	1,2	1,02±0,1	4,4	3,8	4,1	2,4	1,7	3,8±0,4	0,001
VISG4	1,7	1,0	1,0	0,9	1,1	1,06±0,18	92,0	68,4	81,6	21,2	18,6	62,8±21	0,02
CD81	1,4	0,9	0,9	1,0	1,0	1,02±0,1	5,6	3,9	3,8	3,43	4,15	4,3±0,5	0,02
Pmp22	1,4	0,9	0,8	1,1	0,9	1,0±0,1	8,4	12,9	5,8	3,7	3,3	8,6±1,9	0,02
Fcna	1,2	1,2	1,0	1,0	1,0	1,1±0,06	21,3	40,6	19,9	15,1	12,8	23,7±6,9	0,001
HGF	1,0	0,8	0,6	0,9	1,1	0,9±0,2	2,1	1,6	4,2	2,1	2	2,3±0,6	0,057
EGF	1,4	0,8	0,6	1,2	0,6	1,06±0,19	1,3	0,8	1,5	1,2	0,6	1,1±0,2	ns
VEGFA	1,2	0,8	0,5	1,0	1,0	1,1±0,2	0,55	0,5	1,35	0,96	1,09	1±0,2	ns

C

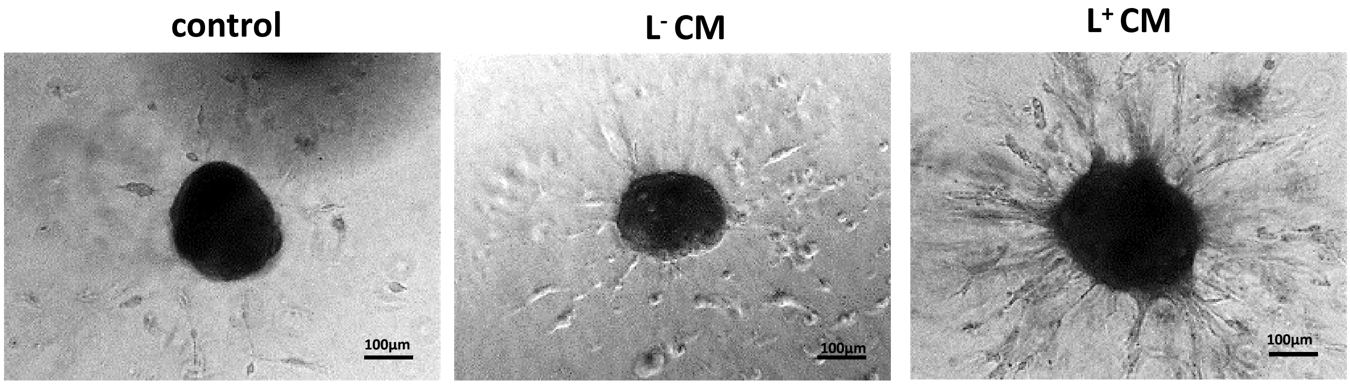

D

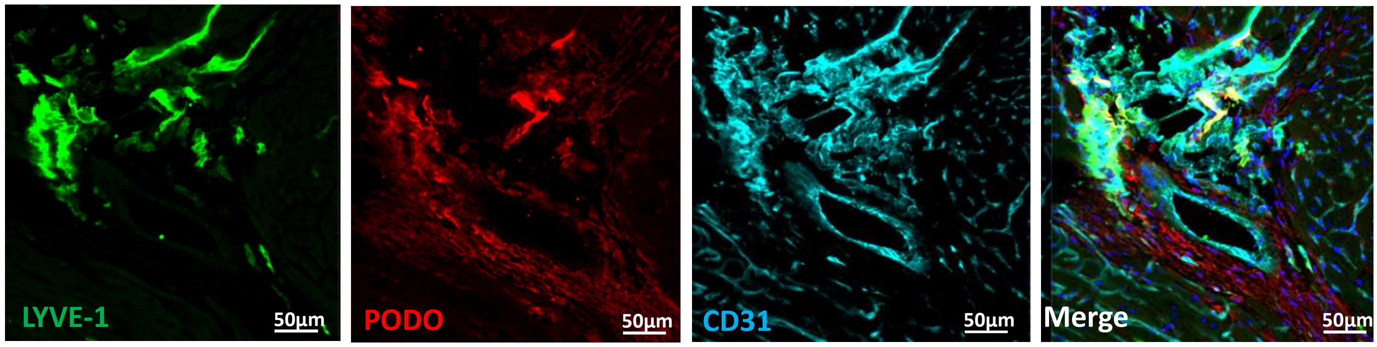

Supplementary Figure 6

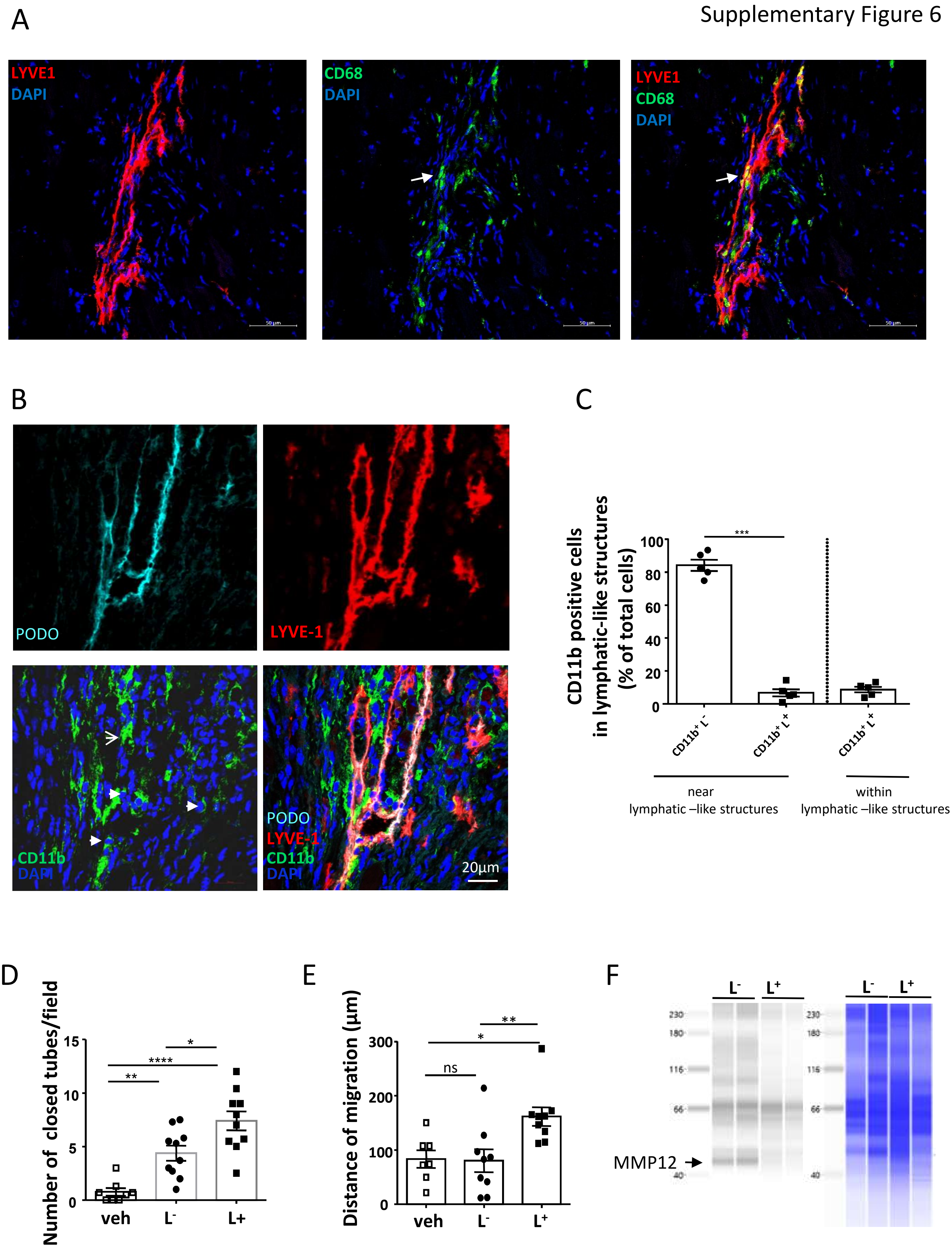

Supplementary Figure 7

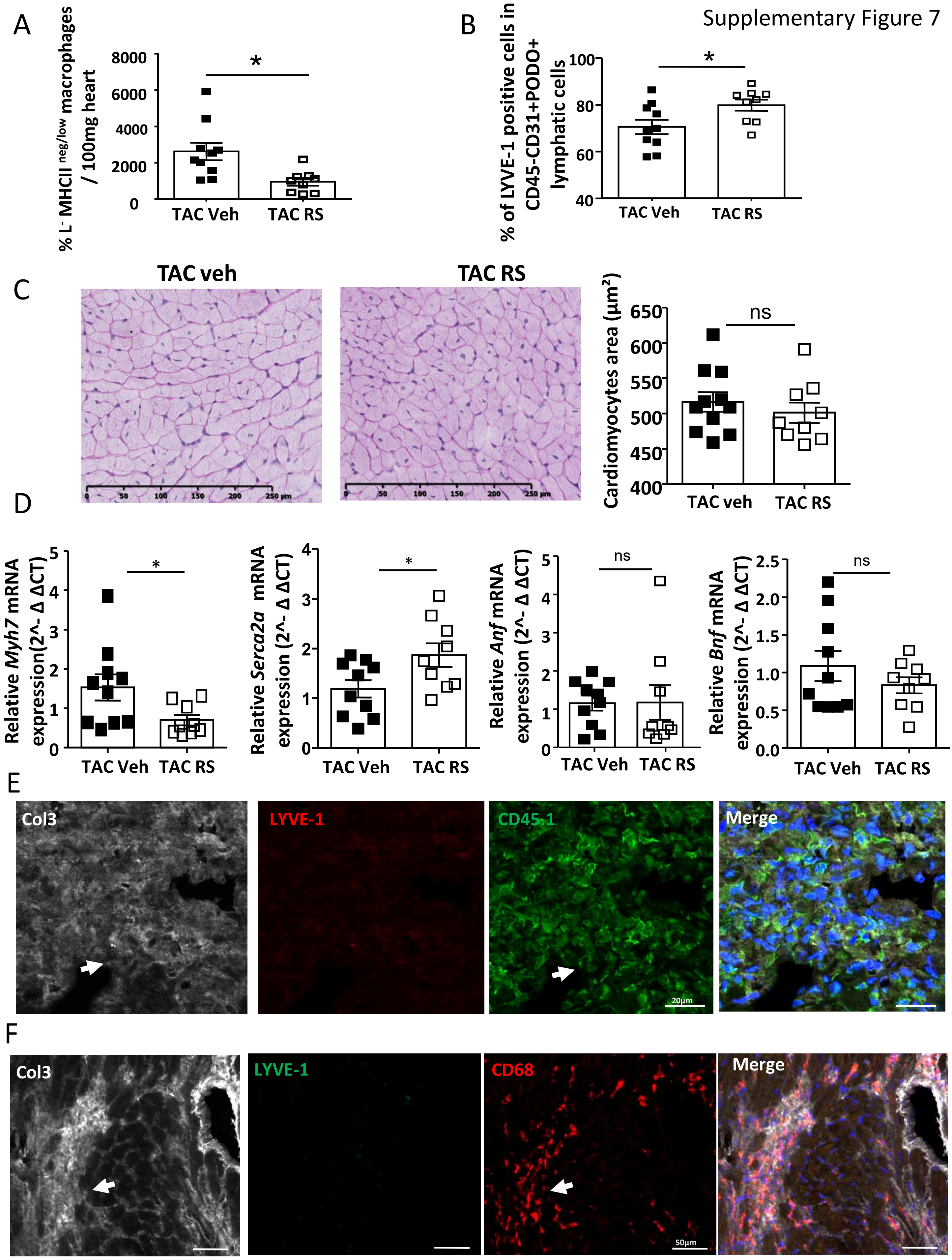

Supplement: Supplementary file 2 — Supplementary Figures. [file 41598_2021_95723_MOESM2_ESM.pdf]
