# Supplementary material for: Cardiac macrophage subsets differentially regulate lymphatic network remodeling during pressure overload
Source: Sci Rep. 2021 Aug 19;11:16801. doi: 10.1038/s41598-021-95723-y (PMC8376913; doi:10.1038/s41598-021-95723-y)
Supplement: Supplementary file 3 [file 41598_2021_95723_MOESM1_ESM.odt]

Cardiac macrophage subsets differentially regulate lymphatic network remodeling during pressure overload

Mathilde Bizou,1 Romain Itier,1,2 Mina Majdoubi,1 Dounia Abbadi,1 Estelle Pichery 1, Marianne Dutaur,1 Dimitri Marsal,1 Denis Calise,3 Barbara Garmy-Susini 1, Victorine Douin-Echinard,1 Jérome Roncalli,1,2 Angelo Parini,1 Nathalie Pizzinat,1

Supplementary methods and figures 

Supplementary methods

Immunohistology, Immunofluorescence and Histological Staining.

Cryosections of 7µm of cardiac tissues frozen in OCT tissue embedding compound (Tissue Tek, EMS) at -80°C or whole hearts were fixed in 4% PFA and blocked using the Avidin/Biotin blocking kit (Vector Labs) followed by 2% normal serum (Sigma-Aldrich) and 1% BSA in PBS. Sections were immunostained overnight with a panel of the following antibodies: rabbit anti Ki-67 (sigma), rabbit anti LYVE-1 (Fitzgerlad), rat anti LYVE-1 (clone ALY; ebiosciences), rat anti CD68 (clone FA-11; AbDSerotec), hamster anti podoplanin (clone 8.1.1;ebiosciences) and rat anti CD31 (BD Pharmingen), and goat anti VEGFR3 (R&D), followed by staining with appropriated secondary antibodies and DAPI. For whole heart, background peroxidase was blocked with peroxidase blocking buffers from Dako) and LYVE-1 staining was revealed by using Vectastain Elite ABC- HRP  Kit (Vector) and colorimetric detection was visualized using DAB substrate. Images were acquired by a binocular microscope SMZ800 (Nikon). Sirius red staining was performed on 10µm cryosections as previously described[4](#_ENREF_4). Periodic acid Schiff (PAS) staining was used to measure cross sectioned cardiomyocytes size. Slides were fixed by immersion in Carnoy’s fixative, rinsed and incubated with periodic acid 0.5% for 10min, then Schiff’s reagent was added on slides for 45 min. Nuclei were stained with hematoxylin and then slides were washed and dehydrated before mounting. Slides were digitized with a Hamamatsu NanoZoomer. Finally, stained areas and total surface from each section were determined using color-based thresholding and quantified by ImageJ software. Fibrotic areas were calculated as percentage of the slice total myocardial area. The area of 50 cardiomyocytes per heart was measured with NDP.view2 software (Hamamatsu).

Bone marrow transplantation and Determination of Reconstitution Efficiency

One day before transplantation, C57Bl/6 mice housed in ventilated cages were treated with 0.17mg/ml of baytril (Bayer, France) in water. The animals received 9Gy total body irradiation. On the day after, bone marrow (BM) cells isolated from CD45.1 C57Bl/6 mice were injected intravenously with 8.106 cells/ mouse to rescue the hematopoietic system of the irradiated CD45.2 mice. Eight weeks after the transplantation, complete engraftment of donor BM in recipients was verified by flow cytometry and mice were submitted to TAC procedure. After 1 week of TAC, heart was collected as described previously {Laroumanie, 2014}.  

Supplemental Table 1: Antibodies used for flow cytometry and FACS procedures

| Antibody | Clone | Provider |
| --- | --- | --- |
| Anti CD45 | 30F11 | BioLegend |
| Anti CD11b | M1/70 | BioLegend |
| Anti CD31 | 390 | BioLegend |
| Anti IA/IE(MHCII) | M5/114.15.2 | BioLegend |
| Anti CD64 | X54-5/7.1 | BioLegend |
| Anti LYVE-1 | ALY7 | Ebiosciences |
| Anti CD206 | MCA2235F | BioLegend |
| Anti CD86 | GL1 | BioLegend |
| Anti Ly6G | RA3-6B2 | BioLegend |
| Anti Ki67 | 145-2C11 | BioLegend |
| Anti podoplanin | 8.1.1 | Ebiosciences |

Real time PCR analysis

RNA was extracted using the Qiagen RNeasy Mini Kit from cardiac tissue, according to the manufacturer’s instructions. DNase treatment was systematically performed. Quality and quantity of extracted RNA were assessed by Experion analysis. The cDNA was synthesized using Superscript II First-Strand system or RT-Vilo (Invitrogen). The absence of contaminants was checked by RT-PCR assays of negative control samples in which the Superscript II was omitted. mRNA was analyzed by real-time PCR using SYBR green (Takara) probe method and the primers listed in Supplemental Table 2. Melting curve analysis was performed to ensure purity of the PCR products and relative quantification was calculated using the comparative cycle threshold (CT) method (2-∆∆CT) with data normalized to housekeeping genes 36B4 and HPRT and calibrated to the average of control group (SHAM unless otherwise notified).

Supplemental Table 2: Primer sequences used for PCR analysis

| Symbol |  | gene |
| --- | --- | --- |
| ANF | F:AAG-GCC-AAG-ACG-AGG-AAG-AAG | [NM_008725.2](http://www.ncbi.nlm.nih.gov/nucleotide/142378163?report=genbank&log$=nuclalign&blast_rank=23&RID=RD6P1U0P01R) |
| R: AGA-GTG-GGC-AGA-GAC-AGC-AAA |
| CCR2 | F: CCT-TGG-GAA-TGA-GTA-ACT-GTG-TGA | NM_009915.2 |
| R:AAT-GAC-AGG-ATT-AAT-GCA-GCA-GTG-T |
| FLT3 | F:ATCTCCCAATTGCACGGAGG | NM_010229.2 |
| R:GAGTACTGCTCGACACCCAC |
| MYH7 | F: TGA-GCC-TTG-GAT-TCT-CAA-ACG-T | [NM_080728.2](http://www.ncbi.nlm.nih.gov/nucleotide/118131045?report=genbank&log$=nucltop&blast_rank=8&RID=RD708GCE01R) |
| R: AGG-TGG-CTC-CGA-GAA-AGG-AA |
| VEGFR3 | F:CCA-TCG-AGA-GTC-TGG-ACA-GC | NM_008029.3 |
| R: CCG-GGA-TGG-TGG-TCA-CAT-AG |
| MERTK | F:AAG-GTC-CCC-GTC-TGT-CCT-AA | NM_008587.1 |
| R:GCG-GGG-AGG-GGA-TTA-CTT-TG |
| CD163 | F:CAT-CAT-GGC-ACA-GGT-CAC-CC | NM_145137.2 |
| R:CGC-TGA-ATC-TGT-CGT-CGC-TT |
| TNFa | F: TGT CCA GGGCAA GCT CAT CT, | [NM_011332.3](http://www.ncbi.nlm.nih.gov/nucleotide/225735578?report=genbank&log$=nucltop&blast_rank=3&RID=RD7JN920014) |
| R: ATG CCT CAG CGG GAA GGT |
| NRP2 | F:TGC-ATG-GAG-TTC-CAG-TAC-CA | NM_001077403.1 |
| CCC-TAT-CAC-TCC-CTC-GAA-CA |
| CD206 | F: TGT CCA GGGCAA GCT CAT CT, | [NM_011332.3](http://www.ncbi.nlm.nih.gov/nucleotide/225735578?report=genbank&log$=nucltop&blast_rank=3&RID=RD7JN920014) |
| R: ATG CCT CAG CGG GAA GGT |
| YM1 | F: AAT-GAT-TCC-TGC-TCC-TGT-GG, | NM_009892.2 |
| R: ACT-TTG-ATG-GCC-TCA-ACC-TG |
| ARG1 | F : TCA CCT GGT GGTGGT CAA CT, | NM_007482.3 |
| R: GGG GCC CAG TTC TTA AAG CC |
| HPRT | F :TGAAAGACTTGCTCGAGATGTCAT | NM_013556.2 |
| R: TCCAGCAGGTCAGCAAAGAA |
| 36B4 | F : GCT-TCA-TTG-TGG-GAG-CAG-AC | NM_007475.5 |
| R : ATG-GTG-TTC-TTG-CCC-ATC-AG |
| IL-10 | F: CCA-AGC-CTT-ATC-GGA-AAT-GAT-C | NM_010548.2 |
| R: CAC-CCA-GGG-AAT-TCA-AAT-GCT |
| Il1beta | F: CTG CAC TAC AG GCT CCG AGA T | NM_008361.3 |
| R: TGT TGG TTG ATA TTC TGT CCA TTG A |
| NLRP3 | F:GCC-CGA-GAA-AGG-CTG-TAT-CC | NM_145827.3 |
| R: CGT-GTC-ATT-CCA-CTC-TGG-CT |
| VEGFc | F: CAT GCA GTT GTT ACA GAA GAC CG | NM_009506.2 |
| R: GGA CAC AGC GGC ATA CTT CT |
| VEGFd | F: CCT ATT GAC ATG CTG TGG GAT | NM_010216.2 |
| R: GTG GGT TCC TGG AGG TAA GAG |
| VEGFa | F :GGC GAG GCA GCT TGA GTT AA | NM_001025250.3 |
| R:TGG TTC CCG AAA CCC TGA G |
| IGF-1 | F: CAA-AAG-CAG-CCC-GCT-CTA-TC | NM_001111274.1 |
| R:CTT-CTC-CTT-TGC-AGC-TTC-GT |
| FGF2 | F: GGC TGC TGG CTT CTA AGT GT | NM_008006.2 |
| R:GTC CCG TTT TGG ATC CGA GT |
| PMP22 | F:GTT-GCT-CTT-CGT-CTC-CAC-CA | NM_008885.3 |
| R:CAA-GGC-GGA-TGT-GGT-ACA-GT |
| FCNA | F:GGC-CTT-TTC-AGG-ACT-GCT-CT | NM_007995.3 |
| R:CCC-CCA-GAC-CTA-CGA-CCT-TA |
| C1qc | F:AAG-GAC-GGG-CAT-GAT-GGA-CT | NC_000070.6 |
| R: CTT-CTG-ACC-CTT-GGG-TCC-TC |
| Lyve1 | F: GGC-TTT-GAG-ACT-TGC-AGC-TAT-G | NM_053247.4 |
| R: GCA-GGA-GTT-AAC-CCA-GGT-GT |
| MMP12 | F: TGC-AGC-AGT-TCT-TTG-GGC-TA | NM_008605.3 |
| AGA-TGC-TGT-ACA-TCG-GGC-AC |
| MMP8 | F: CTTGCCCATGCCTTTCAACC | NM_008611.4 |
| TCATGAGCAGCCACGAGAAA |
| MMP2 | F: CTG-ATG-GCC-CCG-ATC-TAC-AC | NM_008610.2 |
| AGC-TCC-TGG-ATC-CCC-TTG-AT |
| MMP14 | GCCCTCTGTCCCAGATAAGC | NM_008608.3 |
| CCAGAACCATCGCTCCTTGA |
| MMP9 | F: CGC-TCA-TGT-ACC-CGC-TGT-AT | NM_013599.4 |
| GCC-TTG-GGT-CAG-GCT-TAG-AG |
| Vsig4 | CTA-TGG-AGT-GAG-CAG-GGT-GTT | NM_177789.4 |
| GCG-ATG-GTA-GTC-ACC-CTT-GT |

Supplementary Figure legends:

# Supplementary Figure 1: A: Representative images of immunofluorescent staining of LYVE-1 (green) in macrophages identified by CD68 (red) in the heart of WT mice. Merged image illustrates coexpression, and nuclei were stained by DAPI (blue). Scale bar, 25 µm. B-C: Flow cytometry quantification of efferocytosis in freshly isolated cardiac macrophage subsets showing the percentage of positive cells for CFSE labeled cardiomyocytes. Data are presented in mean±SEM (B). Flow cytometry dot plots of the relative amount of cardiomyocytes ingested by the different subsets measured by the CFSE positive cells (C). 

# Supplementary Figure 2: A: Flow cytometry quantification of CD45+Ly6G−CD11b+CD64+L-MHCIIneg macrophage subsets isolated from cardiac tissue after one week of TAC or SHAM. Data are presented in mean ± SEM. Significance vs SHAM: *P<0.05, by t test. B: Representative images of immunofluorescent staining of LYVE-1 (green) and CD68 (red) in the LV of heart after one weeks of TAC or SHAM.  Scale bar, 250 µm. C:  Flow cytometry contour plots showing the MHCII and LYVE-1 expression in macrophages (CD11b+ and CD64high cells) after 6 weeks of TAC or SHAM. D: Flow cytometry quantification of CD45+Ly6G−CD11b+CD64+L-MHCIIneg macrophage subsets isolated from cardiac tissue after six weeks of TAC or SHAM. Data are presented in mean ± SEM. Significance vs SHAM: **P<0.01, by t test.

# Supplementary Figure 3: A: Expression of LYVE-1 (white), podoplanin (red) and VEGFR3 (green) in cardiac subepicardial lymphatic vessels of mouse. (DAPI in blue, Scale bar: 100µm). B: Representative image of lymphatic vessels in left ventricle wall of SHAM mouse heart showing immunofluorescent staining for CD31, LYVE-1 and podoplanin. Open arrow indicates a lymphatic vessel positive for CD31 and podoplanin and with low LYVE-1 staining. Closed arrows indicate a lymphatic vessel negative for podoplanin and positive for CD31 and LYVE-1 (scale bar, 250µm, DAPI in blue). C: Flow cytometry gating strategy for the identification of the different cells populations isolated from hearts, one or six weeks after TAC or sham operations. Cells were first identified on a forward scatter/side scatter (FSC-A/SSC-A) dot plot, the doublets were removed. In live cell populations, the CD45-CD31+LYVE-1- cells were identified as vascular endothelial cells and separated from other  stromal cells. Representative histograms of LYVE-1 mean fluorescence intensity (MFI) in CD45-CD31+ PODO+ cells after 6 weeks of SHAM or TAC. 

Supplementary Figure 4: A: Whole mount staining of heart with anti-LYVE-1 showing a rarefaction of subepicardial lymphatic vessels after one week of TAC. (scale bar, 2mm) and squares show enlarged images of subepicardial lymphatic vessels B: Quantification by flow cytometry of CD45-CD31+LYVE-1+ lymphatic endothelial cells found in cardiac tissue after one week of SHAM or TAC (flow gating strategy detailed in supplementary Fig. 3C). Data are presented in mean ± SEM. Significance  **P<0.01; by t-test. C: The number of CD45-CD31+LYVE-1+ lymphatic cells found in cardiac tissue of each individual mouse after six weeks of TAC was positively correlated with their LV fractional shortening. rs=0.64, p= 0.028 by Spearman. The line comes from a linear regression model.

Supplementary Figure 5. A: Flow cytometry gating strategy used for the identification and isolation of macrophages from Freund’s incomplete adjuvant induced lymphangioma. B: Excel was used to create a heatmap representation of color-coded expression of genes differentially expressed in L- and L+ subsets isolated from five different induced lymphangioma. mRNA expression of gene was normalized to L- subset and p value was determined by Mann-Whitney test. The mean±SEM correspond to RT-PCR quantification of 6 L- (L-Mhigh) and 10 L+ macrophage isolations.. C: Representative images of lymphatic duct explants embedded in type I collagen gel and cultured for 10 days in conditioned medium of lymphangioma isolated L- or L+ macrophages or control media (Scale bar: 100µm). D: Confocal images of immunofluorescent staining of CD31, LYVE-1 and podoplanin in lymphatic-like structures formed in heart one month after the injection of 200 000 L+ macrophages. Scale bar: 50µm.

# Supplementary Figure 6. A: Confocal images of immunofluorescent CD68 staining (green) in LYVE-1 positive lymphatic-like structures (red) formed one month after the cardiac injection of 200 000 L+ macrophages. Few CD68 positive cells were found in the lining of newly formed lymphatic like capillaries present in heart after L+ macrophages injection. (Scale bar: 50µm). B-C: Confocal images of immunofluorescent CD11b staining (green) in LYVE-1 (red) and podoplanin (cyan) positive lymphatic-like structures formed one month after the cardiac injection of 200 000 L+ macrophages (B). Profuse CD11b positive cells (monocytes and L- macrophages) were found surrounding lymphatic-like structures (open arrow) whereas CD11b and L+ positive cells were scarce (closed arrows).  In addition, few CD11b and L+ positive cells were stained within the lining of newly formed lymphatic-like vessels (Scale bar: 20µm). C: Quantification of CD11b positive staining distribution near and within lymphatic-like structures. Data are presented in mean ± SEM. ***P<0.001, by t test. D: Quantification of closed tube-like structures formed by HDLECs when plated on growth factor-reduced Matrigel-coated tissue culture plates and incubated with conditioned medium of L- or L+ macrophages isolated from lymphangioma. *P<0.05; **P<0.01; ****P<0.0001 by Kruskal-Wallis test. E: Quantification of migration in wound healing assay of HDLECs incubated with conditioned medium of L- or L+ macrophages isolated from lymphangioma. Total distance of migration was measured by the difference between the two time points and reported. Data are presented in mean ± SEM. Significance *P<0.05; **P<0.01; by ANOVA test. ns indicates not significant.  F:  Full-lengths capillaries obtained for MMP12 immunodetection in lymphangioma isolated L- and L+ macrophages (left panel) and quantification of loaded proteins (right panel) 

  

Supplementary Figure 7: 

A: Flow cytometry quantification of CD45+Ly6G−CD11b+CD64+L-MHCIIneg macrophage subsets isolated from heart after one week of TAC of mice treated with either vehicle (TAC veh) or RS504393 (TAC RS). Data are presented in mean ± SEM. Significance **P<0.01; by t test. B: Percentage of CD45-CD31+PODO+ endothelial lymphatic cells positive for LYVE-1 in heart after one week of TAC of mice treated with either vehicle (TAC veh) or RS504393 (TAC RS). Data are presented in mean ± SEM. Significance *P<0.05; by t test. C: Representative images of Periodic acid Schiff (PAS) staining of heart after TAC from mice treated with either vehicle (TAC veh) or RS504393 (TAC RS) and quantification of cardiomyocytes size. Data are presented in mean ± SEM. D: Relative expression of gene associated with cardiac remodeling in heart of mice submitted to one week of TAC and treated with either vehicle (TAC veh) or RS504393 (TAC RS). mRNA expression of gene was normalized to TAC control. ns indicates not significant by t test. Relative expression of gene associated with cardiac remodeling in heart of mice submitted to one week of TAC and treated with either vehicle (TAC veh) or RS504393 (TAC RS). mRNA expression of gene was normalized to TAC control, *P<0.05; by t test. E: Representative confocal images of immunofluorescent staining of collagen III (white), LYVE-1 (red) and CD45.1 (green) in the left ventricular myocardium of CD45.2 mice transplanted with bone marrow of CD45.1 mice one week after TAC. Arrow shows that LYVE-1 positive staining was absent in collagen III deposition areas that was infiltrated with hematopoietic CD45.1 cells. (Scale bar: 20µm).  F: Representative confocal images of immunofluorescent staining of collagen III (white), LYVE-1 (green) and the macrophage marker CD68 (red) in the left ventricular wall of mouse heart after one week of TAC. Arrow shows that collagen III deposition accumulated L- macrophages. (Scale bar: 50µm).
